# Ultra-Performance Liquid Chromatography-Mass Spectrometry-Based Untargeted Metabolomics Reveals the Key Potential Biomarkers for Castor Meal-Induced Enteritis in Juvenile Hybrid Grouper (*Epinephelus fuscoguttatus*♀ × *E*. *lanceolatus*♂)

**DOI:** 10.3389/fnut.2022.847425

**Published:** 2022-06-16

**Authors:** Kwaku Amoah, Xiao-hui Dong, Bei-ping Tan, Shuang Zhang, Shu-yan Chi, Qi-hui Yang, Hong-yu Liu, Xiao-bo Yan, Yuan-zhi Yang, Haitao Zhang

**Affiliations:** ^1^Laboratory of Aquatic Animal Nutrition and Feed, College of Fisheries, Guangdong Ocean University, Zhanjiang, China; ^2^Aquatic Animals Precision Nutrition and High-Efficiency Feed Engineering Research Centre of Guangdong Province, Zhanjiang, China; ^3^Key Laboratory of Aquatic, Livestock and Poultry Feed Science and Technology in South China, Ministry of Agriculture, Zhanjiang, China

**Keywords:** *Epinephelus fuscoguttatus*♀ × *Epinephelus lanceolatus*♂, fishmeal, metabolomics, ricinine, castor meal, scanning electron microscopy, ultra-performance liquid chromatography-mass spectrometry (UPLC-MS), partial least-squares discriminant-analysis (PLS-DA)

## Abstract

The intensification of aquaculture to help kerb global food security issues has led to the quest for more economical new protein-rich ingredients for the feed-based aquaculture since fishmeal (FM, the ingredient with the finest protein and lipid profile) is losing its acceptability due to high cost and demand. Although very high in protein, castor meal (CM), a by-product after oil-extraction, is disposed-off due to the high presence of toxins. Concurrently, the agro-industrial wastes’ consistent production and disposal are of utmost concern; however, having better nutritional profiles of these wastes can lead to their adoption. This study was conducted to identify potential biomarkers of CM-induced enteritis in juvenile hybrid-grouper (*Epinephelus fuscoguttatus*♀ × *Epinephelus lanceolatus*♂) using ultra-performance liquid chromatography-mass spectrometry (UPLC-MS) alongside their growth and distal intestinal (DI) health evaluation. A total of 360 fish (initial weight = 9.13 ± 0.01g) were randomly assigned into three groups, namely, fish-meal (FM) (control), 4% CM (CM4), and 20% CM (CM20). After the 56-days feeding-trial, the DI tissues of FM, CM4, and CM20 groups were collected for metabolomics analysis. Principal components analysis and partial least-squares discriminant-analysis (PLS-DA, used to differentiate the CM20 and CM4, from the FM group with satisfactory explanation and predictive ability) were used to analyze the UPLC-MS data. The results revealed a significant improvement in the growth, DI immune responses and digestive enzyme activities, and DI histological examinations in the CM4 group than the others. Nonetheless, CM20 replacement caused DI physiological damage and enteritis in grouper as shown by AB-PAS staining and scanning electron microscopy examinations, respectively. The most influential metabolites in DI contents identified as the potential biomarkers in the positive and negative modes using the metabolomics UPLC-MS profiles were 28 which included five organoheterocyclic compounds, seven lipids, and lipid-like molecules, seven organic oxygen compounds, two benzenoids, five organic acids and derivatives, one phenylpropanoids and polyketides, and one from nucleosides, nucleotides, and analogues superclass. The present study identified a broad array of DI tissue metabolites that differed between FM and CM diets, which provides a valuable reference for further managing fish intestinal health issues. A replacement level of 4% is recommended based on the growth and immunity of fish.

## Graphical Abstract

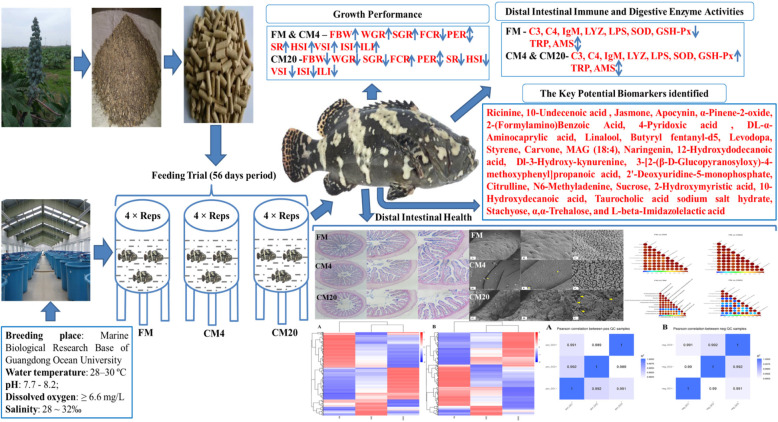

## Introduction

With reference to the highlights of the global food security and nutrition (1), there are about 11% (over 820 million people) of the world’s population (majority from south Asia and sub-Saharan Africa) that remain undernourished, a little up from the 10.6% in 2015. The direct causes emanate from increasing population, income inequalities, conflicts, instability, poverty, and ineffective nutrition policies. Over the last two decades, the aquaculture industry has expanded rapidly to produce highly nutritious food at a relatively lower price (2). The FAO, in their 2020 report, revealed that, despite the decline in aquaculture production, the sector played a major role in the 179 million tonnes of the global fish produced, of which 156 million tonnes were used for human consumption (3). However, the aquaculture industry in increasing production to meet the global demands has been faced with lots of challenges including the over-exploitation of natural stocks, perishability, and food-borne diseases. Market globalisation and recurring food-safety alerts have resulted in increasing consumer awareness. In the last decade, the seafood sector has incentivised the application of relevant novel molecular techniques for the monitoring and assessment of food safety, traceability, and quality due to the perishable nature of seafood and the key role they play as a protein source for the global population (4, 5). Thus, regarding the development of the global economy and the upgrading of living standards, humans are calling for higher quality seafood, and the problem of fish health deterioration and quality instigated by feed has become one of the central problems that cannot be disregarded in the aquaculture industry today. The increasing demand for fish products further intensifies the supply pressure of feed materials, especially fishmeal (FM) which is mainly sourced from marine capture fisheries. FM is the preferred source of protein in aquaculture because of its high protein content, balanced essential amino acids (EAA), high palatability, and digestibility (6). Among the other animal food-producing sectors, the aquaculture industry is noted as the largest consumer of FM because the industry consumes about 68–78% (7, 8). The current shortfall of marine capture fisheries has drastically increased the cost of FM as demand exceeds the supply (3). Exploring alternative conventional feeds such as plant-based protein (PBP) diets is a necessity as they are less costly and readily accessible (9, 10). However, the extensive research and documentation of most of the PBP ingredients, including soybean meal (SBM), cottonseed meal, peanut meal, corn gluten meal, wheat gluten meal, and others used in substituting FM partially or fully are gradually making them costly and not accessible enough as farmers’ acceptance rates and demands keep on increasing (9–13). There is therefore an urgent need for the assessment of other PBPs that are readily available in larger quantities and can substitute FM.

Castor meal (CM) is a by-product generated after the extraction of oil from the castor plant (*Ricinus communis* L.) seed (14). Hitherto, the consistent production and safe discarding of agro-industrial wastes have been a matter of concern since their natural oxidation directly fortifies the quantum of greenhouse gases creating awful effects on human and animal health. CM has the potential of being used as a protein supplement due to its high protein (35–55.8% dry matter depending on the seed characteristics) and energy (32–49% dry matter) levels as compared to other PBP ingredients (15, 16). A comparative study of the amino acid compositions of CM revealed an almost similar pattern for all EAAs in SBM (mostly substituted PBP for FM) except lysine and sulphur amino acids (17). Castor meal-induced enteritis (CMIE) like other PBP-induced enteritis (18, 19) refers to the non-infectious subacute enteritis, and histological characteristics such as shortened mucosal folds, lamina propria and submucosa swelling, infiltration of various inflammatory cells, and reduced absorption of intestinal epithelial cells after dietary supplementation of CM. The intestine is an important organ that suffers lots of pathogenic microorganism effects and toxic damages (20, 21). Generally, the available evidence postulates that the effects of CM on intestinal health of animals are related to the imbalanced amino acids and the anti-nutritional factors (ANFs) such as ricin, ricinine, allergen, agglutinins, tannins, lectin, oxalate, and phytases contained in CM (22–25). Thus, CM is being precluded from being used as an animal feed supplement. Previously conducted studies show that CM raw material can usually be added to feed at a relatively low level since excessive addition to diets may not only reduce feed intake and growth but also affect the intestinal structure and cause enteritis in the gut of animals (16, 26–30). Although there are very limited studies on the supplementation effects of CM in fish, the studies conducted on hybrid catfish (*Hetero clarias*) exceeding 12.5% inclusion (31) and juvenile grass carp (*Ctenopharyngodon idella*) exceeding 5% inclusion (32) all show a reduction in growth performance, feed utilisation, and body composition, unless CM raw material is reduced or detoxified. However, there are lack of systematic studies on the effect of effects of CM on intestinal enteritis in fish.

In understanding the effects of aquaculture feeds on the physiology of fish, there must be a shift in the methods used to discover diet and intestinal health relations from different standpoints. The use of new omics technologies is noted to show enormous potential to aid in the understanding of the complex interplay between the nutrition and immunity of fish (33). As one of the newest “omics” sciences, metabolomics deals with the supplementation of data from transcriptomics, genomics, and proteomics to promote the understanding of biological systems (34). Metabolomic studies provide great potential insight into biomarker identification, diagnosis of diseases, and toxicological mechanism (35, 36) since they can reveal the changes and laws of endogenous metabolites in an organism after external interference (37). Currently, metabolomics has been extensively used in drug discovery and food safety fields, thus, becoming an effective tool to investigate the biochemical effects of toxic substances (38), which warrants more research.

The hybrid grouper (*Epinephelus fuscoguttatus*♀ × *E*. *lanceolatus*♂), is one of the most sought after fish in China and the world and is currently used for intensive and super-intensive aquaculture as a result of their enormous attributes such as faster growth, efficient food utilisation, higher resistive capacity to disease infection, being able to withstand higher population density (39, 40), higher nutritional and market value (41, 42), aside from their ability to adapt to high salinity conditions (43). As a typical carnivore marine new species, the hybrid grouper’s dietary protein requirement ranges between 50 and 55% (44). FM is the primary protein source for grouper, but this fish is less adapted to PBP diets due to utilisation and enteritis problems (45).

This study aimed at identifying differential metabolites linked with dietary CM by conducting untargeted metabolomics using ultra-performance liquid chromatography-mass spectrometry (UPLC-MS). Notably, there is non-existence of studies correlating dietary CM supplementation with their metabolite profiling in fish. For the first time, this study demonstrates the effects of replacing FM with CM on growth, feed utilisation, immune response, digestive enzyme activities, and histological examination in hybrid grouper. Furthermore, our study’s main attraction and novelty are premised on the fact that we have been able to identify candidate biomarkers of the overall CM pattern, which is a more comprehensive perspective given that nutrients do not act in isolation.

## Materials and Methods

### Experimental Diets

All of the procedures were performed following the relevant policies of Animal Welfare in China. The Animal Research and the Ethics Review Board of Guangdong Ocean University approved the animal protocol used in the present study. The FM used in the current study was supplied by China National Township Enterprise, whereas the CM was purchased from the Shangdong Weifang Supply and Marketing Industrial Co. Ltd. (Shangdong, China). Three iso-nitrogenous (approximately 50% crude protein), and iso-lipidic (approximately 10% of total lipid) experimental diets were formulated to contain 0, 4.76, and 23.79% of CM by replacing 0% (FM, control), 4% (CM4), and 20% (CM20) of FM protein. The Supplementary Table 1 illustrates the amino acid profiles of the FM and CM ingredients used in this study. The ingredient formulation composition and the proximate chemical analysis of the experimental diets are presented in Table 1. Crystalline amino acid (AA) methionine, lysine, threonine, and leucine were added to the diets to achieve the required amino acid for grouper feed. The balancing of AA profiles during the formulation was done in strict accordance with previously reported work (46).

**TABLE 1:** Formulation and proximate composition of experimental diets (% dry matter).

Ingredients	FM	CM4	CM20
Red fish meal^a^	50	46	30
Castor meal^b^	0	4.76	23.79
Wheat gluten meal^c^	9	9	9
Soy protein concentrate^d^	7	7	7
Wheat Flour^c^	16	16	16
Casein^e^	2	2	2
Corn oil^c^	2	2	2
Fish oil^c^	2.5	2.8	4
Soy lecithin^c^	1.5	1.5	1.5
Vitamin premix^f^	0.5	0.5	0.5
Mineral premix^g^	0.5	0.5	0.5
Choline chloride^h^	0.5	0.5	0.5
Vitamin C^a^	0.05	0.05	0.05
Ca(H_2_PO_4_)_2_^h^	1	1	1
Attractant^a^	0.1	0.1	0.1
Ethoxyquin^a^	0.05	0.05	0.05
Microcrystaline cellulose^i^	6.8	5.53	0.47
Carboxymethyl cellulose^i^	0.5	0.5	0.5
Methionine^j^	0	0.04	0.19
Lysine^j^	0	0.12	0.6
Threonine^j^	0	0.02	0.08
Leucine^j^	0	0.03	0.16
Total	100	100	100
**Proximate composition**			
Crude protein	50.00	49.98	49.43
Crude lipid	10.10	10.57	10.03
Moisture	7.10	7.13	7.88
Ash	12.58	12.18	11.71

*^a^Red fish meal: crude protein, 70.03%, and crude lipid 8.24% (supplied by China National Township Enterprises Corporation).*

*^b^Castor meal: crude protein, 58.87% and crude lipid, 0.42% (purchased from Shandong Weifang Supply and Marketing Industrial Co., Ltd., Shandong, China).*

*^c^Wheat gluten meal: crude protein, 81.22%, and crude lipid, 0.11%; Wheat flour: crude protein, 10.52%, and crude lipid, 0.36%; corn oil; fish oil; soy lecithin; vitamin C; (purchased from Zhanjiang Haibao Feed Co. Ltd., Guangdong, China).*

*^d^Soy protein concentrate: crude protein, 67.87% and crude lipid, 0.46% (supplied by Shandong Changrun Biology Co., Ltd).*

*^e^Casein: crude protein, 92.43% and crude lipid, 0.11 (purchased from Sigma Chemical Co., Ltd., Shanghai, China).*

*^f^Vitamin premix (g kg^–1^ mixture): vitamin B_1_, 17.00 g; vitamin B_2_, 16.67 g; vitamin B_6_, 33.33 g; vitamin B_12_, 0.07 g; vitamin E, 66.00 g; vitamin K, 3.33 g; vitamin D, 33.33 g, retinyl acetate, 6.67 g; D-calcium pantothenate, 40.67 g; nicotinic acid, 67.33 g; folic acid, 4.17 g; biotin, 16.67; inositol, cellulose, 592.72 g; and 102.04 g (obtained from Zhanjiang Yuehua Feed Co. Ltd., Zhanjiang, China).*

*^g^Mineral premix (g kg^–1^ premix): ZnSO_4_.H_2_O, 32.0991 g; FeSO_4_^⋅^7H_2_O, 18.785 g; MgSO_4_.H_2_O, 65.19927g, CoCl_2_.6H_2_O (10%), 5.5555g; CuSO_5_.5H_2_O, 11.0721 g; KIO_3_, 0.0213 g; Na_2_SeO_3_ (10%), 0.5555 g, KCl, 22.7411 g; zeolite powder, 843.9777 g (Obtained from Zhanjiang Yuehua Feed Co. Ltd., Zhanjiang, China).*

*^h^Purchased from Shanghai Macklin Biochemical Co. Ltd., Shanghai, China.*

*^i^Purchased from Shantou Xilong Chemical Factory, Guangdong, China.*

*^j^These amino acids were added to balance the amino acid content in the FM (control) diet. Purchased from Shanghai Doublewin Bio-Tech. Co., Ltd.*

The proximate chemical analysis of the experimental diets followed standardised methods of AOAC (47). Briefly, the moisture content was determined by drying feed in an oven at 105°C until a constant weight was obtained. The crude lipid was determined by the Soxhlet method using ether extraction. Also, while the crude protein (N × 6.25) was determined by the method of Kjeldahl, which involves the use of an Auto Kjeldahl System (8400-Autoanalyzer, FOSS), the ash content was analysed by muffle furnace combustion involving oven incineration at 550°C for 5 h. In preparing the diets, all the ingredients were ground and sieved (60 mesh size sieve). Afterward, they were gradually mixed with fish oil, corn oil, and soybean lecithin, and finally, purified water was added to make dough. The dough was then pelleted through a double helix extrusion machine (F-75 pelletizer, South China University of Technology, China). The pellets made (2 and 2.5 mm diameter) were air-dried, and kept in sealed Ziploc bags, which were subsequently stored at –20°C till the commencement of the feeding trial. The EAA and non-essential amino acid (NEAA) contents in the various experimental diets are shown in the Supplementary Table 2.

### Experimental Fish and Culture Condition

Healthy hybrid grouper (*Epinephelus fuscoguttatus*♀ × *E*. *lanceolatus*♂) were obtained from a commercial farm (Zhanjiang, Guangdong Province, China). Upon arrival, all fish were acclimatised in aerated cement pools (4.5 m [L] × 3.45 m [W] × 1.8 m [H]) for 2 weeks. Fish were hand-fed twice daily with a commercial diet during the acclimatisation period. Afterward, 360 juvenile fish of uniform size were starved for 24 h. They were then weighed and randomly distributed into 12 cylindrical fibreglass tanks (0.5 m^3^) at 30 fish densities per tank. They were hand-fed during the 8-weeks experiment period to apparent satiation twice daily (08:00 and 16:30) with the three experimental diets (FM, CM4, and CM20). The experiment was conducted at an indoor facility of the Marine Biological Research Base of Guangdong Ocean University (China) for 56 days. Single-airstones provided water aeration. During the feeding period, the water quality parameters were maintained daily by renewing 30% of the filtered seawater for the first 2 weeks, which shifted to 50% renewal to keep the temperature, pH, dissolved oxygen, and salinity within the ranges 28–30°C, 7.7–8.2, ≥6.6 mg L^–1^, and 28–32‰, respectively (YSI 556 multiprobe system, YSI Inc., United States). The photoperiod was 12 h L: 12 h D, with the light period from 7:30 am to 7:30 pm.

### Sampling of Fish

#### The Performance of Growth

At the termination of the experiment, all fish after 24 h starvation period were anaesthetised with eugenol (1:10,000) before harvest. The total numbers of fish left per individual tank were counted, and their mean body weights were taken. Three fish were randomly sampled to record their body and intestinal lengths, liver, and intestinal weights. Based on the obtained records, the growth performance parameters, including the survival rate (SR), weight gain rate (WGR), specific growth rate (SGR), feed conversion ratio (FCR), hepatosomatic index (his), viscerosomatic index (VSI), intestinal somatic index (ISI), and intestinal length index (ILI) were calculated as described below;


(1) $$SR,\%=100\times \frac{Finalfishnumber}{Initialfishnumber};$$

(2) $$WGR,\%=100\times \frac{Finalfishbodyweight\left(g\right)-Initialfishbodyweight\left(g\right)}{Initialfishbodyweight\left(g\right)};$$

(3) $$\begin{array}{c} \;SGR,\%=100\times \\ \frac{\text{ln}\left[Finalfishbodyweight\left(g\right)\right]-\ln\left[Initialfishbodyweight\left(g\right)\right]}{Daysofexperiment}; \end{array}$$

(4) $$FCR=\frac{Totaldryfeedintake\left(g\right)}{Finalfishbodyweight\left(g\right)-Initialfishbodyweight\left(g\right)};$$

(5) $$HSI,\%=100\times \frac{Fishliverweight\left(g\right)}{Fishbodyweight\left(g\right)};$$

(6) $$VSI,\%=100\times \frac{Fishvisceraweight\left(g\right)}{Fishbodyweight\left(g\right)};$$

(7) $$ISI,\%=100\times \frac{Finalfishintestineweight\left(g\right)}{Finalfishbodyweight\left(g\right)};and$$

(8) $$ILI,\%=100\times \frac{Finalfishintestinelength\left(cm\right)}{Finalfishbodylength\left(\mathrm{cm}\right)}.$$

#### Distal Intestinal Sampling for Histological Examination and Metabolomics Analysis

To aid in the analysis of intestinal enzyme activity, two fish from each tank were randomly selected and their intestines removed. The intestines were cleared of any mesenteric adipose tissue and rinsed with deionised water. The cleaned distal intestines (DI) were cut, placed in Eppendorf tubes, and immediately frozen in liquid nitrogen. Samples were later stored at –80°C for subsequent enzyme activity analysis.

Eight fish from each treatment group (two fish per replicate tank) were randomly selected and their intestines removed for the scanning electron microscopy (SEM) analysis. The removal of the DI of the fish was done within 1–3 min, and the tissue mass was not more than 3 mm. The bloodstains and other tissues on the sample surface were removed by gently washing with PBS (pH 7.4) for the 30 s followed by fixing in a 2.5% glutaraldehyde (purchased from Wuhan Servicebio Technology Co. Ltd., Wuhan, China). The samples were kept for 24 h (at 4°C) until further processing. For Alcian Blue-Periodic Acid-Shiff (AB-PAS) staining analysis, a fish per replicate tank was randomly selected to remove the DI tissue samples, which were immediately placed in a 4% formaldehyde solution and stored for subsequent analysis.

For metabolomics analysis, six fish were randomly sampled from each treatment group to get the DI samples. The samples were instantaneously frozen in liquid nitrogen and stored at –80°C for subsequent analysis.

### Determination of Enzyme Activities

After removing the frozen intestinal samples, they were thawed, weighed, and homogenised in 0.9% sterilised saline at 1:9 (*tissue*:*volume*) with the help of a bead homogeniser in ice for 10 min. The homogenate was later centrifuged (3000 × *g* for 15 min at 4°C). The supernatant was collected in 1.5 mL tubes and used the determination of enzyme activities. The immune and antioxidant titres of the intestine, including lysozyme (LYZ), Immunoglobulin M (IgM), complement 3 (C3) and 4 (C4), superoxide dismutase (SOD), and glutathione peroxidase (GSH-Px) activities, were determined using the fish LYZ Elisa detection kit, fish IgM Elisa detection kit, fish C3 and C4 Elisa detection kit, fish SOD Elisa detection kit, and fish GSH-Px Elisa detection kit, respectively. The kits were all procured from the Shanghai Jianglai Biotechnology Co. Ltd. (Shanghai, China).

Following the manufacturer’s protocol, to a flat-bottomed 96-well plate pre-coated with fish LYZ antibody, 50 μl each of the standard or intestinal sample (sample final dilution is fivefolds) solution was added to the appropriate wells and mixed gently without touching the good wall. Subsequently, 100 μl of horseradish Peroxide (HRP)-conjugate LYZ reagent was added to the sample and standard wells with the exception of blank wells. Plates were covered with closure plates and incubated for 60 min at 37°C. Afterward, liquid in wells was discarded, dried by gently swinging plates, and washed five times (solutions were kept still in wells for 30 s before drying by pat) with wash buffer solution (diluted 20-fold with double distilled water). After plate drying, the 50 μl each of Chromogen Solution A and Solution B were added to all wells and incubated for 15 min at 37°C, and the reaction stopped with 50 μl of stop solution. The optical density (OD) of the LYZ level in each well was read using a Rayto RT-6100 microplate reader (Shenzhen, China) set to 450 nm wavelength. The IgM, C3, and C4 enzymes followed the same procedure as the LYZ, however, fish IgM antibody, fish C3 antibody, and fish C4 antibody were used instead of fish LYZ antibody. The trypsin (TRP) activity was determined according to the methods of Erlanger et al. (48). Lipase (LPS) and amylase (AMS) activities were measured following the methods of Gjellesvik et al. (49) and Yaghoubi et al. (50), respectively. The kits used for the TRP, LPS, and AMS activities were as well purchased from Shanghai Jianglai Biotechnology Co. Ltd. (Shanghai, China). The intestinal enzyme activities were expressed per mg protein concentration (bicinchoninic acid, BCA) (51).

### Distal Intestinal Alcian Blue-Periodic Acid Schiff Section and Scanning Electron Microscopy Analysis

Samples were washed with PBS after removing from the fixation solution and post-fixed by washing tissue blocks with 0.1 M PBS (pH 7.4) three times (15 min per time). The tissue blocks were transferred into 1% osmium tetroxide (OsO_4_) in 0.1 M PBS (pH 7.4) for 1–2 h at room temperature and washed again in 0.1 M PBS (pH 7.4) three times (15 min per each time). Samples were dehydrated in graded ethanol doses (30, 50, 70, 80, 90, 95, and 100% with two baths in 100% ethanol). Isoamyl acetate was used for the final dehydration stage, during which the dehydration time lasted 15 min at each step. Samples were then submitted to critical point drying (Quorum K850, Quorum Tech. Ltd., United Kingdom) and attached to metallic stubs using carbon stickers. Later, they were sputter-coated with gold using MC1000 sputter coater (Hitachi Ltd., Tokyo, Japan) for 30 s. The prepared SEM samples were examined and photographed using an MSIP-REM-htn-SU8100 scanning electron microscope (Hitachi High-Technologies Corporation Corporate Manufacturing Strategy Group, Japan).

For the AB-PAS histological examinations, the tissues removed from the 4% formalin buffer were paraffin-embedded (JB-P5, Wuhan Junjie Electronics Co., Ltd., Wuhan, China), cut into 4μm sections using a microtome (Leica Instruments, Shanghai, China, RM2016). Samples were dewaxed by Xylene I and Xylene II for 20 min each, followed by 100% ethanol I and ethanol II (Servicebio, G1049, Sinaopharm Group Chemical Reagent Co., Ltd., Shanghai, China) for 5 min each, then 75% ethanol for 5 min. Samples were later rinsed with running tap water. Subsequently, the sections were stained with Alcian blue dyes for 15 min. They were rinsed with running tap water till it was colourless and then stained with periodic acid dye for 15 min. Afterward, they were rinsed with running tap water and rinsed twice again with distilled water. They were then placed in Schiff’s reagent and stained again at room temperature for 30 min in the dark followed by rinsing for 5 min. The sections were dehydrated by 100% ethanol I (5 min), 100% ethanol II (5 min), 100% ethanol III (5 min), Xylene I (5 min), Xylene II (5 min), and later sealed with the neutral gum. The images were then captured as previously described (52, 53) with Olympus model BX51 (Serial number: 9K18395, Tokyo, Japan). The villi height (VH), villi width (VW), crypt depth (CD), lamina propria (LP), and intestinal epithelial muscle thickness (MT) were measured using the software Image-Pro Plus 6.3 (Media Cybernetics, Inc., Rockville, MD, United States). The goblet cell (GC) counts were measured using cellSens Standard 1.8 software.

### Analysis of Metabolomics

#### Metabolites Extraction and Ultra-Performance Liquid Chromatography-Mass Spectrometry Analysis

The individual DI samples (100 mg) were ground with liquid nitrogen, and their homogenate was re-suspended using prechilled 80 and 0.1% formic acid by the good vortex. Subsequently, the samples were incubated on ice (5 min), centrifuged (15,000 × *g* for 20 min at 4°C), and aliquots of supernatant samples were diluted to a final concentration containing 53% methanol by LC-MS grade water. The samples were consequently transferred into fresh Eppendorf tubes (with 0.22 μm), centrifuged (15,000 × *g* for 20 min at 4°C), and finally, the filtrate was injected into the LC-MS/MS system analysis (54). UPLC-MS/MS analyses were performed using a Vanquish UPLC system (Thermo Fisher, Germany) coupled with an Orbitrap Q Exactive™ HF-X mass spectrometer (Thermo Fisher, Germany) at Novogene Co., Ltd. (Beijing, China). Samples were injected onto a Hypesil Gold column (100 × 2.1 mm, 1.9 μm) using a 17 min linear gradient at 0.2 ml/min flow rate. The eluents for the positive polarity mode were eluent A (0.1% FA in Water) and eluent B (Methanol). The eluents for the negative polarity mode were eluent A (5 mM ammonium acetate, pH 9) and eluent B (Methanol). The solvent gradient was set as follows: 2% B, 1.5 min; 2–100% B, 12 min; 100% B, 14.0 min; 100-2% B, 14.1 min; 2% B, 17 min. Q Exactive™ HF-X mass spectrometer was operated in positive/negative polarity mode with a spray voltage of 3.2 kV, capillary temperature of 320°C, sheath gas flow rate of 40 arb, and aux gas flow rate of 10 arb.

#### Quality Control

As metabolomics is easily disturbed by external factors and changes rapidly, data quality control (QC), which can detect anomalies in time, is necessary to obtain stable and accurate metabolome results. In controlling the quality of the experiment conducted while sample processing, QC samples were prepared. The QC samples are the equal mixing samples of treatment samples used to balance the chromatographic-mass spectrometry system, monitor the LC-MS system performance state, and evaluate the system’s stability during the whole experiment process. Based on the relative quantitative value of metabolites, the Pearson correlation coefficient between QC samples was calculated. The higher the correlation of QC samples (the closer to 1), the better the stability of the whole method. Again, the distribution of QC samples in the PCA analysis diagram can be termed as having better stability of the whole method and that there is a higher data quality when QC samples are reflected as being smaller and are clustered together (55). At the same time, blank samples are set up to aid in the removal of background ions.

#### Processing of Data and Metabolite Identification

The UPLC-MS/MS raw data generated were processed using Compound Discoverer 3.1 (CD 3.1, Thermo Fisher) to perform a peak alignment, peak picking, and quantitation of each metabolite. The main parameters were set as follows: retention time tolerance, 0.2 min; actual mass tolerance, 5 ppm; signal intensity tolerance, 30%; signal/noise ratio, 3; and minimum intensity, 100,000. Subsequently, peak intensities were normalised to the total spectral intensity. The normalised data were used to predict the molecular formula based on additive ions, molecular ion peaks, and fragment ions. The peaks were then matched with the *mz*Cloud^1^, mzVault, ChemSpider^2^, and MassList databases to obtain accurate qualitative and relative quantitative results. Statistical analyses were then performed using the statistical software R (R version R-3.4.3), Python (Python 2.7.6 version), and CentOS (CentOS release 6.6). When data were not normally distributed, normal transformations were attempted using the area normalisation method. The metabolites were annotated using the Kyoto Encyclopedia of Genes and Genomes (KEGG) database^3^, Human Metabolome Database (HMDB)^4^ and LIPID MAPS^®^ database^5^. Later on, the principal component analysis (PCA) and partial least squares discriminant analysis (PLS-DA) was performed using a flexible and comprehensive software for metabolomics processing known as meta X software. An application of univariate analysis (*t*-test) was made to calculate the statistical significance (*P*-value). The Variable Importance in the Projection (VIP) value reflects the contribution of each variable to the model. Larger VIP values were considered as the major potential biomarkers for differentiating the control and the experimental groups. The metabolites with VIP > 1 and *P* < 0.05 and fold change ≥ 2 or FC ≤ 0.5 were considered as differential metabolites (56).

#### Differential Metabolites’ Filtering

The Venn plot of differential metabolites between the DI tissues was made to search for the co-contained differential metabolite, that is, the overlapping part in the Venn plot. The Log2FC values of the differential metabolites in the coinciding part were calculated, and the co-contained differential metabolites changed with contrary trends and were considered to be significantly affected after ingestion by fish. The left of the co-contained metabolites with the same trend and differential times of Log2FC < 2 were considered no-significantly changed and removed in the whole differential metabolites of the DI contents. Volcano plots were used to filter metabolites of interest-based on log2FC and –log10 (*p*-value) of metabolites. For heatmap clustering, the data were normalised using *z*-Scores of the intensity areas of differential metabolites and were plotted by Pheatmap package in R language. The correlation between differential metabolites was analysed by cor.mtest function in the R-package (method = Pearson). The statistically significant correlation between differential metabolites was calculated by cor.mtest in the R-package where the threshold level of significant correlation was *P* < 0.05. The functions of these metabolites and metabolic pathways were studied using the KEGG database. The metabolic pathways enrichment of differential metabolites was performed. When the ratio was satisfied by x/n > y/N, metabolic pathways were considered enriched, and when the *P*-value of the metabolic pathway was < 0.05, metabolic pathways were regarded as statistically significantly enriched.

### Statistical Analysis

The statistical analyses were done using the Statistical Package for Social Sciences (SPSS) for Windows software (IBM SPSS version 20, Inc., 2010, Chicago, IL, United States). To examine the differences between groups, a one-way analysis of variance (ANOVA) was conducted when the data variance was homogenous. Differences were considered statistically significant at *P* < 0.05 between treatment groups using Tukey’s Honest Significant Difference (HSD) tests. Receiver operator characteristic (ROC) curve analysis was conducted for metabolites to determine the Area Under Curve (AUC), which compares the predictive ability of metabolites.

## Results

### Growth Performance

As illustrated in Table 2, the CM4 group used in replacing the FM group experienced significantly high (*P* < 0.05) final body weight (FBW), WGR, SGR, HSI, VSI, ISI, and ILI. In contrast, the FCR was significantly higher (*P* < 0.05) in the CM20 group than in the CM4 and FM groups. No significant difference (*P* > 0.05) was observed in the PER and SR between all groups.

**TABLE 2:** Effects of different levels of castor meal substitute for fish meal protein on the growth performance, immune and digestive enzyme activities of juvenile hybrid grouper (*Epinephelus fuscoguttatus*♀ × *E*. *lanceolatus*♂).

Parameters	FM	CM4	CM20
**Growth**			
IBW (g)	9.12 ± 0.01	9.13 ± 0.00	9.13 ± 0.00
FBW (g)	80.44 ± 1.31^b^	85.07 ± 0.87^b^	56.21 ± 3.10^a^
WGR (%)	781.99 ± 14.67^b^	832.02 ± 9.53^b^	515.59 ± 33.76^a^
SGR (% day^–1^)	3.89 ± 0.03^b^	3.92 ± 0.07^b^	3.24 ± 0.10^a^
FCR	0.78 ± 0.03^a^	0.74 ± 0.01^a^	0.93 ± 0.02^b^
PER	2.90 ± 0.10	2.86 ± 0.10	2.59 ± 0.14
SR (%)	98.33 ± 1.68	95.00 ± 2.16	95.57 ± 4.43
**Morphological**			
HSI (%)	2.12 ± 0.16^ab^	2.42 ± 0.13^b^	1.71 ± 0.09^a^
VSI (%)	9.06 ± 0.15^b^	9.55 ± 0.16^b^	7.63 ± 0.49^a^
ISI (%)	0.63 ± 0.02^a^	0.78 ± 0.03^b^	0.59 ± 0.04^a^
ILI (%)	144.44 ± 2.45^b^	147.45 ± 2.62^b^	126.48 ± 3.06^a^
**Immune**			
C3 (μg mgprot^–1^)	103.67 ± 2.83^a^	148.72 ± 14.81^b^	120.08 ± 8.27^ab^
C4 (μg mgprot^–1^)	222.22 ± 5.05^a^	265.04 ± 13.54^ab^	292.14 ± 12.16^b^
IgM (μg mgprot^–1^)	94.47 ± 1.11^a^	101.27 ± 1.14^b^	106.34 ± 1.29^c^
LYZ (U gprot^–1^)	6.18 ± 0.50^a^	13.71 ± 0.93^b^	17.90 ± 1.29^c^
SOD (ng mg.prot^–1^)	9.61 ± 0.34^a^	13.37 ± 1.25^b^	17.65 ± 0.94^c^
GSH-Px (ng mg.prot^–1^)	27.21 ± 1.78^a^	40.58 ± 1.98^b^	47.84 ± 2.38^b^
**Digestive enzymes**			
TRP (U mgprot^–1^)	1868.71 ± 110.84	2317.29 ± 171.15	2156.58 ± 107.61
LPS (U mgprot^–1^	120.56 ± 10.75^a^	474.57 ± 38.91^c^	279.67 ± 25.23^b^
AMS (U mgprot^–1^)	299.25 ± 13.47	382.55 ± 35.30	380.11 ± 18.10

*Data are mean values of four replicates ± SE. The means in the same line with no superscript letters do not differ significantly among groups (P > 0:05) based on Tukey’s HSD test. Where: IBW, initial body weight; FBW, final body weight; WGR, weight gain rate; SGR, specific growth rate; FCR, feed conversion ratio; PER, protein efficiency ratio; SR, survival rate; HSI, hepatosomatic index; VSI, viscerosomatic index; ISI, intestinal somatic index; ILI, intestinal length index; C3, complement 3; C4, complement 4; IgM, Immunoglobulin M; LYZ, lysozyme; SOD, superoxide dismutase; GSH-Px, glutathione peroxidase; TRP, trypsin; LPS, lipase; and AMS, amylase; FM, fish meal (control group); CM4, 4% CM protein replacement to FM protein; CM20, 20% CM protein replacement to FM protein.*

### Immune, Antioxidant, and Digestive Enzyme Indices

The results of the DI immune and digestive enzyme indices are shown in Table 2. The C3 activity was significantly higher in the CM4 group than in the other groups. On the other hand, CM’s replacement levels in fish diets significantly increased (*P* < 0.05) the C4, IgM, LYZ, SOD, and GSH-Px immune and antioxidant enzymes concentrations, with the CM20 groups witnessing the highest value. No significant differences (*P* > 0.05) were observed among all groups concerning the TRP and AMS digestive enzymes. Nonetheless, a significantly elevated (*P* < 0.05) LPS activity was observed in the CM4 and CM20 groups than in the FM group, with the CM4 revealing the highest activity.

### Distal Histological Examination

**FIGURE 1:**
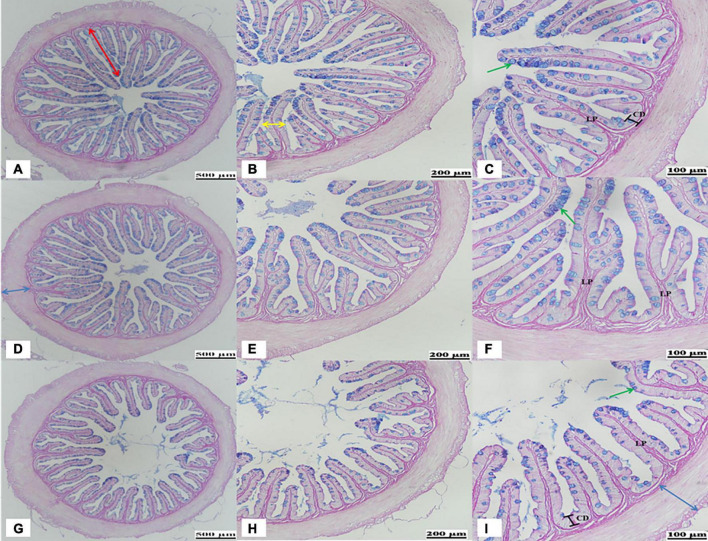
Representative histological evaluation of the distal intestine in hybrid grouper fed the FM **(A–C)**, CM4 **(D–F)**, and CM20 **(G–I)** diets based on Alcian Blue-Periodic Acid-Schiff (AB-PAS) staining. **(A,D,E)** Shows a decreasing villi height (red arrows) and increased mucosal folds (blue arrows) as replacement levels increases. **(B,C,E,F,H,I)** Shows the changes in the villi width (yellow arrow), lamina propria (LP), crypt depth (CD), and goblet cells (green arrows). FM, fish meal (control group); CM4, 4% castor meal (CM) protein replacement to FM protein; CM20, 20% CM protein replacement to FM protein.

Castor-meal substitution in the diet caused a significant change in the histological analysis of the DI. The results obtained regarding the DI histology examined by AB-PAS staining are presented in Figure 1 and Table 3. It was revealed after the 56-day feeding trial that the 20% CM replacement level led to a significant reduction (*P* < 0.05) in the villi height, villi width, crypt depth, and goblet cells. On the other hand, a significant increase (*P* < 0.05) in muscle thickness and lamina propria width was observed in the CM20 group in comparison to the FM and CM4 groups (Figure 1 and Table 3).

**TABLE 3:** The distal intestinal tissue morphology of hybrid grouper fed with experiment diets.

Treatment	Villi height (μm)	Villi width (μm)	Muscle thickness (μm)	Crypt depth (μm)	Lamina propria width (μm)	Goblet cells/μm villi height
FM	797.55 ± 9.59*^c^*	128.64 ± 3.76*^b^*	191.79 ± 1.33*^a^*	75.08 ± 1.81*^b^*	14.02 ± 0.69*^a^*	0.06 ± 0.00*^b^*
CM4	730.56 ± 7.94*^b^*	112.40 ± 4.20*^a^*	221.41 ± 4.26*^b^*	80.69 ± 2.64*^b^*	15.06 ± 0.97*^a^*	0.07 ± 0.00*^b^*
CM20	481.55 ± 6.91*^a^*	97.51 ± 3.52*^a^*	309.04 ± 5.70*^c^*	64.91 ± 1.66*^a^*	28.35 ± 1.14*^b^*	0.04 ± 0.00*^a^*

*Data are mean values ± SE. The means in the same column with no superscript letters do not differ significantly among groups (P > 0:05) based on Tukey’s HSD test. Where: FM, fish meal (control group); CM4, 4% castor meal (CM) protein replacement to FM protein; CM20, 20% CM protein replacement to FM protein.*

**FIGURE 2:**
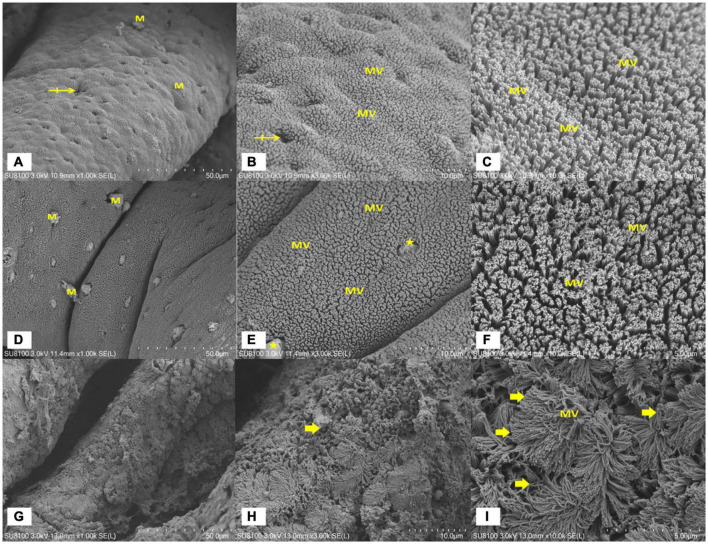
A scanning electron microscopy (SEM) of the distal intestinal mucosal surface of juvenile hybrid grouper fed with FM **(A–C)**, CM4 **(D–F)**, and CM20 **(G–I)**. Bar markers represent 50 μm **(A,D,G)**, 10 μm **(B,E,H)**, and 5 μm **(C,F,I)**. **(A–C)** Shows more and pebbled mucosal surface or villi (MV) density which contains orifices of several goblet cells that may be seen protruding mucus (M) or not (crossed arrow); **(D–F)** shows more and pebbled mucosal surface or villi (MV) density which contains orifices of several goblet cells that may be seen protruding mucus (M) in addition to very few noticeable villi detachments of from the epithelial layer (stars); **(G–I)** shows few and weak mucosal surface or villi (MV) density, irregular orientation of villi, villi atrophy or blunting (flattening) causing some villi to disappear (arrows). Abbreviations are as defined in Figure 1.

As illustrated in Figure 2, the SEM results show significant change among groups. Compared with the other groups, the highest CM replacement group (CM20) was observed to have few and weak mucosal villi density, alongside its irregular villi orientation (Figures 2G–I). Again, there was villi atrophy (flattening or blunting) which in a way led to some of the villi disappearing in contrast to what was observed in the CM4 (Figures 2D–F) and the control group (FM; Figures 2A–C).

### Metabolic Profiling by Ultra-Performance Liquid Chromatography-Mass Spectrometry Analysis

**FIGURE 3:**
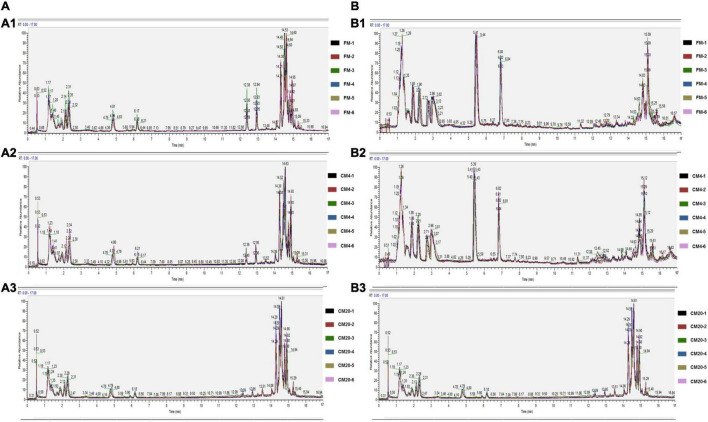
The representative UPLC-MC spectra of the distal intestine in positive **(A)** and negative **(B)** modes. **(A1,B1)** Respectively represent the individual sample repeats of FM in positive and negative modes, **(A2,B2)** respectively represent the individual sample repeats of CM4 in positive and negative modes, and **(A3,B3)** respectively represent the individual sample repeats of CM20 in positive and negative modes. Abbreviations are as defined in Figure 1.

**FIGURE 4:**
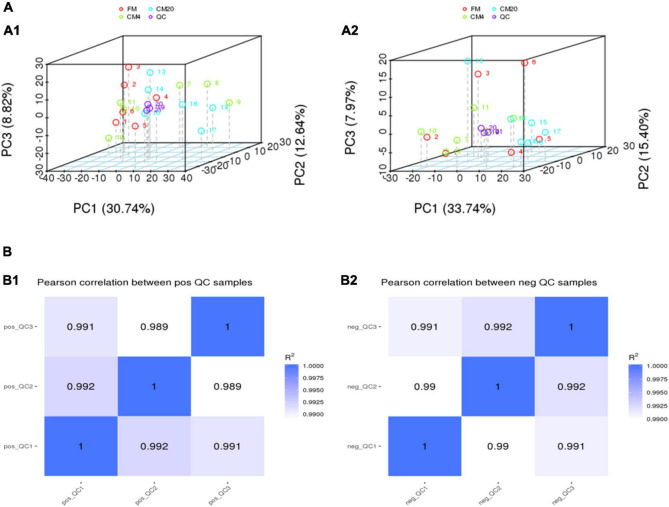
**(A)** The results of the total sample PCA score plots from UPLC-MS spectra of the distal intestinal tissue in the positive **(A1)** and negative **(A2)** modes. Abscissa PC1 and ordinate PC2 represent the scores of the first and second principal components. Scattered dots in different colours represent samples from different experimental groups as illustrated in Figure 3. QC represents the quality control group. **(B)** The QC samples of the distal intestinal contents’ correlation in the positive **(B1)** and negative **(B2)** modes. The abscissa is log10 (Peak. Area + 1), the ordinate (Peak. Area + 1), and the *R*^2^ is the square of the Pearson correlation coefficient. *n* = 6.

Figure 3 and Supplementary Figure 1 show DI content’s representative spectra. It must be noted that the samples of the DI tissue were analysed with UPLC-MS in positive and negative modes. Figure 4A illustrates the score plots of the PCA in the two modes. The QC samples were observed in the centre and were clustered tightly, depicting the better stability of the whole detection process and the higher quality of the data; thus, its sample correlation is shown in Figure 4B.

**FIGURE 5:**
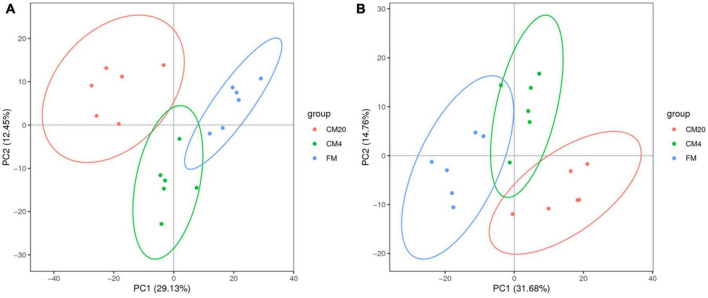
The results of the total PLS-DA score plots from the UPLC-MS spectra of the distal intestinal contents in positive **(A)** and negative **(B)** modes. Blue dots represent the FM group (fishmeal group-control); green dots represent the CM4 groups [4% CM (castor meal) protein replacement to FM protein]; red dots represent CM20 groups (20% CM protein replacement to FM protein). *n* = 6.

**FIGURE 6:**
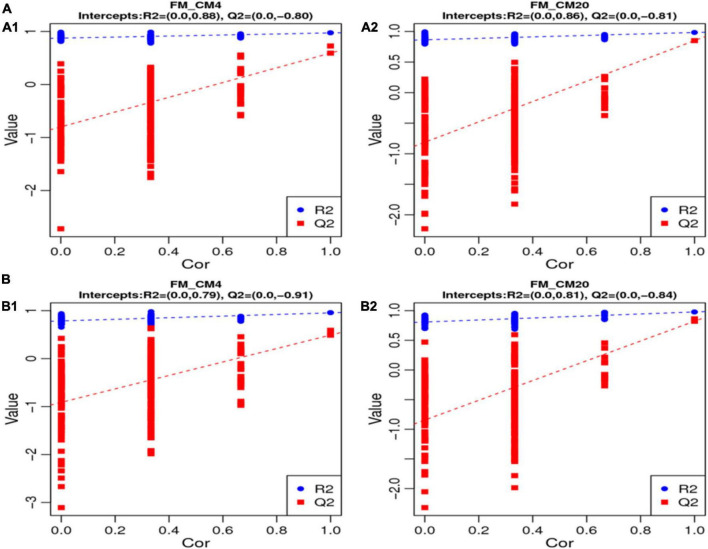
The results of the permutation test of the PLS-DA models of the distal intestinal contents between FM, CM4, and CM20 groups in the positive **(A1,A2)** and negative **(B1,B2)** modes. The R2Y value represents the model’s goodness of fit, whereas the Q2 value represents the predictability of the models. Abbreviations are as defined in Figure 1. *n* = 6.

In characterising the profiles of the metabolites, the Partial Least Squares Discrimination Analysis (PLS-DA) was used, and Figure 5 shows the results of the DI contents in the positive (Figure 5A) and negative modes (Figure 5B). There were clear separations between all groups, and all samples in the respective groups were fundamentally observed to be in the 95% confidence ellipses (Supplementary Figure 2). Consequently, in preventing model over-fitting, permutation tests were conducted in positive and negative modes (Figure 6). The permutation test parameters R2Y and Q2Y in the DI samples’ positive modes were 0.97 and 0.59 between groups of FM and CM4, and 0.98 and 0.85 between groups of FM and CM20. In the negative modes, the permutation test parameters R2Y and Q2Y were 0.96 and 0.49 between groups of FM and CM4, and 0.98 and 0.82 between groups of FM and CM4 of which they were ≥ 0.5. To judge the quality of the model, it is sorted and verified to check whether the model is over-fitting. Model over-fitting reflects the accuracy of the model construction. If the model is not over-fitted, the model can describe the sample well and be used as the premise of searching for the model biomarker group. But if the model is over-fitted, then the model is not suitable for describing the sample and cannot be used for later data analysis. The modelling and prediction are conducted after randomly shuffling the grouping markers of each sample where each of the modellings corresponds to a group of values R2 and Q2 and their regression lines are obtained according to the values of Q2 and R2 after 200 shuffling and modelling. When the R2 data is larger than the Q2 data and the intercept between the Q2 regression line and Y-axis is less than ‘0,’ the model is described as not over-fitted. Thus, the results of the red line (Q2) and the blue line (R2) on the left indicate a low risk of over-fitting the models (Figure 6).

### Differential Metabolites’ Filtering

**FIGURE 7:**
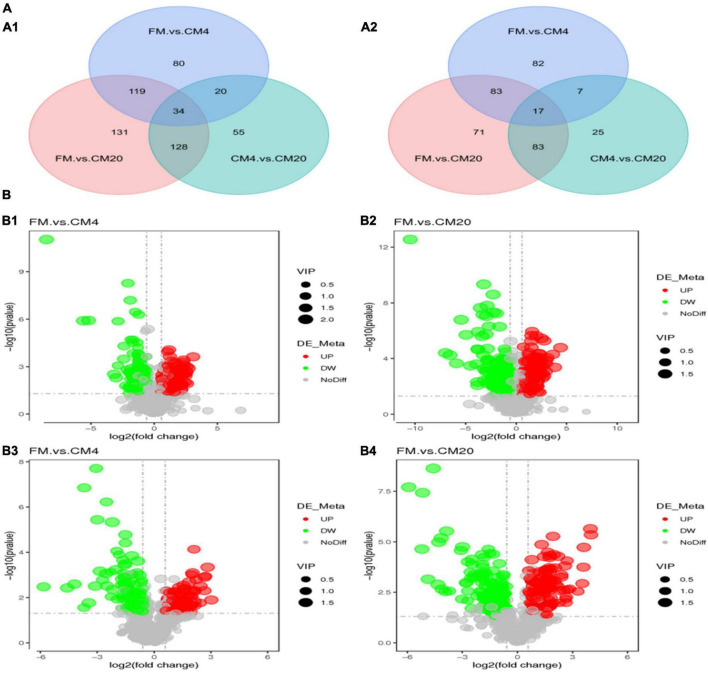
**(A)** The Venn plot displays the differential metabolites of distal intestinal contents of hybrid grouper between experimental groups in positive **(A1)** and negative **(A2)** modes. **(B)** Volcano plots of *p*-values between the FM, CM4, and CM20 groups in the positive **(B1,B2)** and negative **(B3,B4)** modes. The horizontal coordinate represents the change of expression of multiple metabolites in different groups (log2FC), and the vertical coordinate represents the significance level of difference [–log10(*p*-value)]. Each dot in the figure represents a metabolite, and the size of the dot represents the VIP value. The significantly up-regulated metabolites are represented by red dots, the significantly down-regulated metabolites are represented by green dots, and gray dots represent no significantly different metabolites. Abbreviations are as defined in Figure 1. *n* = 6.

Venn diagram is used to display multiple comparative combinations of differential metabolites, which can intuitively compare common and unique differential metabolites between different groups to display the relationship between multiple groups of differential metabolites. From the analysis of the Venn plots, there were 567 differential metabolites in the DI tissue contents, of which 34 were co-contained in positive modes between the various groups. Nonetheless, in the negative modes, 368 differential metabolites were observed in the DI tissue contents, of which 17 were co-contained metabolites between the FM, CM4, and CM20 groups (Figure 7). The Venn plots revealed that most of the differential metabolites observed in the DI tissues for hybrid grouper occurred due to the physiological response of the intestine to diet metabolism. The Supplementary Table 3 illustrates the co-contained differential metabolites in the DI intestine in positive and negative modes.

### Analysis of Differential Metabolite

**FIGURE 8:**
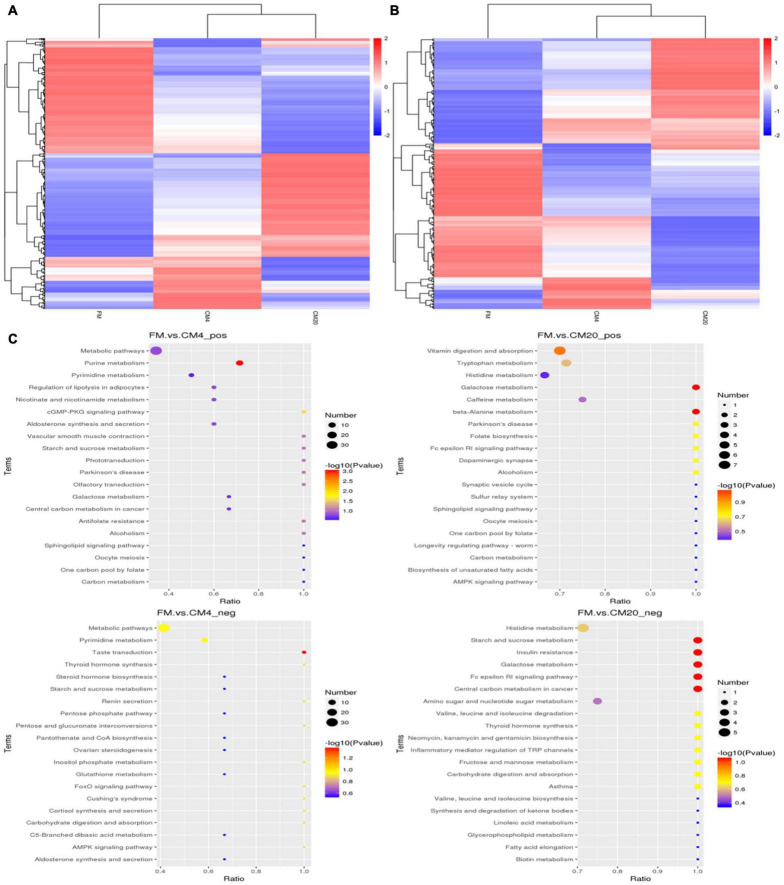
Clustering heatmap of total differential metabolites in the positive **(A)** and negative **(B)** modes after fishmeal replacement with castor meal. Individual groups are clustered in the vertical part, whereas those of the metabolites are clustered in the horizontal part. Colour intensity indicates the intensity of the metabolite. The relationship of metabolite content clustering between groups can be seen horizontally. The shorter the cluster branch is, the higher the similarity is. **(C)** Scatter plot of the top 20 KEGG pathway enrichment for differential metabolites in the FM, CM4, and CM20 groups in positive (pos) and negative (neg) modes. Abbreviations are as defined in Figure 1. *n* = 6.

As illustrated in Figures 7B1,B2, the volcano plot in the positive mode showed significantly that 127 metabolites were up-regulated and 126 metabolites were down-regulated between the FM and CM4 groups (Supplementary Table 4). On the other hand, 213 metabolites were up-regulated and 199 metabolites down-regulated between the FM and CM20 groups (Supplementary Table 5). Again in Figures 7B3,B4, the volcano plot in the negative mode showed significantly that 83 metabolites were up-regulated and 106 metabolites down-regulated between the FM and CM4 groups (Supplementary Table 6). However, 128 metabolites were up-regulated, and 126 metabolites were down-regulated between the FM and CM20 groups in the negative mode (Supplementary Table 7). Hierarchical Clustering Analysis (HCA) was conducted for all the differential metabolites obtained between comparison pairs (57). In the end, the relative quantitative values of differential metabolites were normalised and clustered as presented in Figure 8A (positive mode) and Figure 8B (negative mode) with its details provided in Supplementary Figure 3.

The 567 differential metabolites in the positive modes were enriched into 52 pathways (Supplementary Table 8) between the FM and CM4 groups of which the top 10 pathways were “Purine metabolism,” “cGMP-PKG signalling pathways,” “Starch and sucrose metabolism,” “Antifolate resistance,” “Vascular smooth muscle contraction,” “Olfactory transduction,” “Phototransduction,” “Parkinson’s disease,” “Alcoholism,” and “Metabolic pathways.” On the other hand, there were 64 pathways enriched in positive modes between the FM and CM20 groups (Supplementary Table 9), with the top 10 being “Galactose metabolism,” “beta-Alanine metabolism,” “Vitamin digestion and absorption,” “Folate biosynthesis,” “Fc epsilon RI signalling pathway,” “Dopaminergic synapse,” “Parkinson’s disease,” “Alcoholism,” “Tryptophan metabolism,” and “Caffeine metabolism.” The KEGG-enrichment scatterplot showed that there was only one distinct pathway, “Purine metabolism,” which was significantly enriched (*P* < 0.05) when the FM to CM4 were compared together. In contrast, two distinct pathways, “Galactose metabolism” and “beta-Alanine metabolism,” were significantly enriched (*P* < 0.05) when comparing the FM to CM20 (Figure 8C) in the positive modes.

However, in the negative modes, the 368 differential metabolites were enriched into 60 pathways (Supplementary Table 10) between the FM and CM4 groups of which the top 10 pathways were “Taste transduction,” “Pyrimidine metabolism,” “Metabolic pathways,” “Pentose and glucuronate interconversions,” “Inositol phosphate metabolism,” “FoxO signalling pathway,” “AMPK signalling pathway,” “Thyroid hormone synthesis,” “Renin secretion,” and “Cortisol synthesis and secretion.” On the other hand, there were 64 pathways enriched in negative modes between the FM and CM20 groups (Supplementary Table 11), with the top 10 beings “Galactose metabolism,” “Starch and sucrose metabolism,” “Fc epsilon RI signalling pathway,” “Insulin resistance,” “Central carbon metabolism in cancer,” “Fructose and mannose metabolism,” “Valine, leucine, and isoleucine degradation,” “Neomycin, kanamycin, and gentamicin biosynthesis,” “Inflammatory mediator regulation of TRP channels,” and “Thyroid hormone synthesis.” The KEGG-enrichment scatterplot disclosed that there was only one distinguishing pathway, “Taste transduction,” which was significantly enriched (*P* < 0.05) when the FM to CM4 were compared together, whereas five distinct pathways, “Starch and sucrose metabolism,” “Insulin resistance,” “Galactose metabolism,” “Fc epsilon RI signalling pathway,” and “Central carbon metabolism in cancer,” were significantly enriched (*P* < 0.05) comparing FM to CM20 (Figure 8C) in the negative modes.

Based on the VIP > 1 score, the absolute value of log2(fold change) > 1, and *P* < 0.05, the 10 most influential metabolites differentiating the CM4 from the FM group in the positive mode (Supplementary Table 12) were: “Ricinine (C_8_H_8_N_2_O_2_),” “10-Undecenoic acid (C_11_H_20_O_2_),” “Jasmone (C_11_H_16_O),” “Apocynin (C_9_H_10_O_3_),” “α-Pinene-2-oxide (C_10_H_16_O),” “2-(Formylamino)Benzoic Acid (C_8_H_7_NO_3_),” “4-Pyridoxic acid (C_8_H_9_NO_4_),” “DL-α-Aminocaprylic acid (C_8_H_17_NO_2_),” “Linalool (C_10_H_18_O),” and “Butyryl fentanyl-d5 (C_23_H_25_[2]H_5_N_2_O).” Nevertheless, those differentiating the CM20 from the FM group (Supplementary Table 13) were: “Ricinine (C_8_H_8_N_2_O_2_),” “Jasmone (C_11_H_16_O),” “α-Pinene-2-oxide (C_10_H_16_O),” “Levodopa (C_9_H_11_NO_4_),” “10-Undecenoic acid (C_11_H_20_O_2_),” “Styrene (C_8_H_8_),” “Carvone (C_10_H_14_O),” “Butyryl fentanyl-d5 (C_23_H_25_[2]H_5_N_2_O),” “MAG (18:4) (C_21_H_34_O_4_),” and “DL-α-Aminocaprylic acid (C_8_H_17_NO_2_)” in the positive mode. In the negative mode, the 10 most influential metabolites differentiating the CM4 from the FM group (Supplementary Table 14) were: “12-Hydroxydodecanoic acid (C_12_H_24_O_3_),” “Dl-3-Hydroxy-kynurenine (C_10_H_12_N_2_O_4_),” “Naringenin (C_15_H_12_O_5_),” “3-[2-(β-D-Glucopyranosyloxy)-4-methoxyphenyl]propanoic acid (C_16_H_22_O_9_),” “2′-Deoxyuridine-5-monophosphate (C_9_H_13_N_2_O_8_P),” “Citrulline (C_6_H_13_N_3_O_3_),” “*N6*-Methyladenine (C_6_H_7_N_5_),” “Sucrose (C_12_H_22_O_11_),” “2-Hydroxymyristic acid (C_14_H_28_O_3_),” and “10-Hydroxydecanoic acid (C_10_H_20_O_3_).” However, those differentiating the CM20 from the FM group (Supplementary Table 15) were: “Naringenin (C_15_H_12_O_5_),” “Dl-3-Hydroxy-kynurenine (C_10_H_12_N_2_O_4_),” “12-Hydroxydodecanoic acid (C_12_H_24_O_3_),” “Sucrose (C_12_H_22_O_11_),” “2-Hydroxymyristic acid (C_14_H_28_O_3_),” “Taurocholic acid sodium salt hydrate (C_26_H_45_NNaO_7_S),” “Stachyose (C_24_H_42_O_21_),” “10-Hydroxydecanoic acid (C_10_H_20_O_3_),” “α,α-Trehalose (C_12_H_22_O_11_),” and “L-beta-Imidazolelactic acid (C_6_H_8_N_2_O_3_)” in the negative mode. Given this, a total of 20 metabolites were observed as the key metabolites in the FM and CM4 group (lower replacement level), and 20 key metabolites were also observed in the FM and CM20 group (higher replacement level). However, in analysing the key metabolites, 12 of them were observed in both replacement groups. As a result, a total of 28 metabolites were identified and selected as the key potential biomarkers.

**FIGURE 9:**
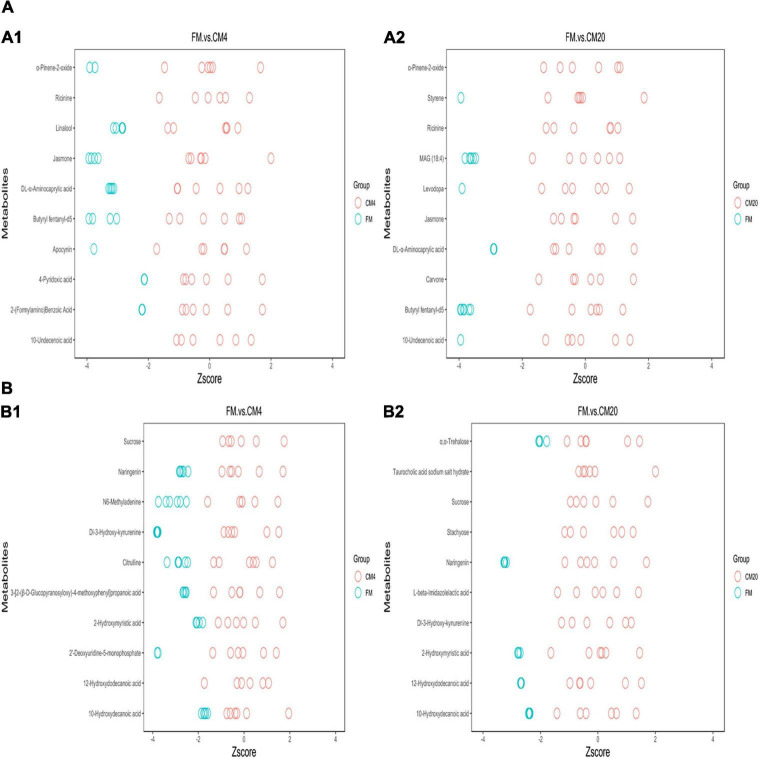
The *z*-Score plot of the potential biomarkers for the comparison between FM, CM4, and CM20 groups in the positive **(A1,A2)** and negative **(B1,B2)** modes. Abbreviations are as defined in Figure 1. *n* = 6.

**FIGURE 10:**
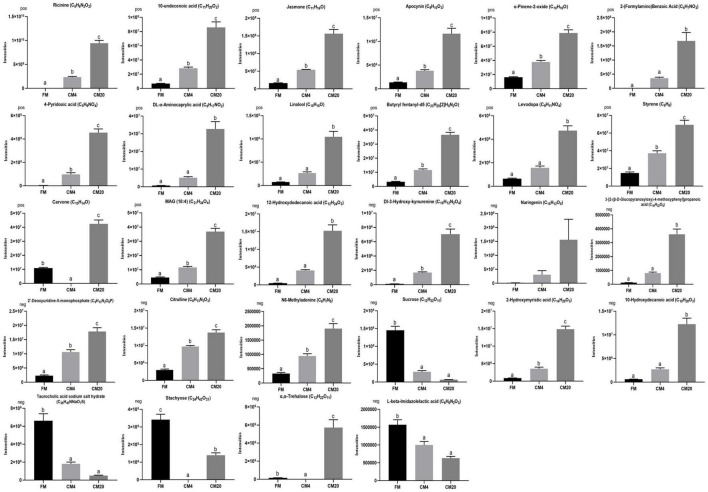
Intensities of the metabolites identified in the positive and negative modes. Data represented the mean ± SE (Turkey’s HSD, *P* < 0.05; *n* = 6). Vertical bars marked with no letters do not differ significantly (*P* > 0.05) among groups. In the bar plots, FM, fishmeal; CM4, 4% replacement of fishmeal with castor meal; and CM20, 20% replacement of fishmeal with castor meal.

Subsequently, *z*-score plots of the metabolites were analysed to define the potential biomarkers further (Figure 9). Figure 10 and Supplementary Tables 16, 17 illustrate the intensities of the potential biomarkers identified in the positive and negative modes. Compared with the FM group, increasing the replacement levels of CM caused a significant increasing trend (*P* < 0.05) in the intensities of Ricinine, 10-Undecenoic acid, Jasmone, Apocynin, α-Pinene-2-oxide, 2-(Formylamino)Benzoic Acid, 4-Pyridoxic acid, DL-α-Aminocaprylic acid, Linalool, Butyryl fentanyl-d5, Levodopa, Styrene, MAG (18:4), 12-Hydroxydodecanoic acid, Dl-3-Hydroxy-kynurenine, 3-[2-(β-D-Glucopyranosyloxy)-4-methoxyphenyl]propanoic acid, 2′-Deoxyuridine-5-monophosphate, Citrulline, *N6*-Methyladenine, 2-Hydroxymyristic acid, and 10-Hydroxydecanoic acid, contrast to as observed in the intensities of Sucrose, Taurocholic acid sodium salt hydrate, Stachyose, and L-beta-Imidazolelactic acid. Nonetheless, no significant differences (*P* > 0.05) were observed between the FM and CM4 groups concerning the intensities of 2-(Formylamino)Benzoic Acid, DL-α-Aminocaprylic acid, Linalool, Levodopa, 12-Hydroxydodecanoic acid, 3-[2-(β-D-Glucopyranosyloxy)-4-methoxyphenyl]propanoic acid, and 10-Hydroxydecanoic acid. Again, for the intensities of Naringenin, no significant difference (*P* > 0.05) was observed among all groups. Interestingly, there were zero intensities of Carvone, Stachyose, and α,α-Trehalose biomarkers observed in the CM4 group. Supplementary Table 18 shows the classification of the 28 differential metabolites identified in the DI tissues of FM vs. CM4 and CM20 groups (HMDB).

**FIGURE 11:**
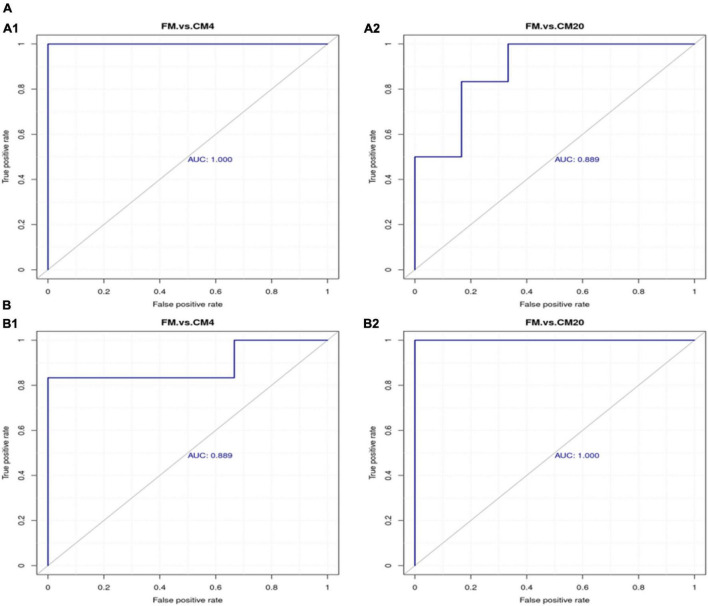
The ROC curve analysis for differentiating among groups with respect to the potential biomarker metabolites in the positive **(A1,A2)** and negative **(B1,B2)** modes. Abbreviations are as defined in Figure 1. *n* = 6.

**FIGURE 12:**
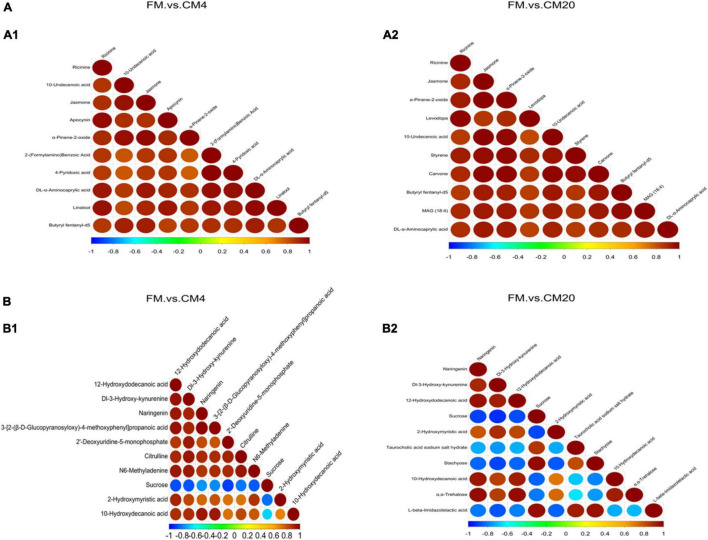
The correlation analysis of the potential biomarkers between FM and CM4 groups and FM and CM20 groups in the positive **(A1,A2)** and negative **(B1,B2)** modes. Abbreviations are as defined in Figure 1. *n* = 6.

The area under the ROC curve is recognised as the area under curve (AUC), which is used to assess the sensitivity and specificity of biomarkers for predicting the occurrence of events (58). The sensitivity and specificity of each metabolite are determined by the optimal threshold of the ROC Curve. Thus, (i) when AUC = 0.5, the biomarker has no predictive value for predicting the occurrence of events, and (ii) when AUC value is > 0.5, the closer it is to 1 depicts the higher the accuracy of prediction. The prediction accuracy is generally low when the AUC value is between 0.5 and 0.7. The prediction accuracy is certain when the AUC value is between 0.7 and 0.9, and the prediction accuracy becomes high when the AUC value is above 0.9. The results of the ROC analysis, as shown in Figure 11, resulted in the AUC of the metabolites exceeding 0.8 at a 95% CI, depicting that there were good predictive abilities for the screened potential metabolite biomarker. The correlation analysis conducted revealed Sucrose, Taurocholic acid sodium salt hydrate, Stachyose, and L-beta-Imidazolelactic acid to have a negative correlation with the other potential metabolite biomarkers, whereas the other potential metabolite biomarkers actively correlated positively with each other (Figure 12).

## Discussion

The present study revealed that the growth performance, intestinal histology, digestion, immune responses, and metabolic profiles identified were significantly changed by dietary replacement of CM. Generally, plant-based proteins (PBPs) are reported to have imbalanced EAA profiles. The inclusion of PBPs in fish diets exposes them to various phytochemicals, including antinutritional factors (ANFs), which interfere with nutrient digestibility, absorption, and utilisation and ultimately affect the growth performance and health status (9, 10, 17). CM also contains ANFs such as ricin, ricinine, allergen, agglutinins, tannins, lectin, oxalate, and phytases, as well as low levels of EAA, including lysine and methionine (22–25, 59). As a carnivorous fish, the demand for protein and amino acids for grouper must be of high quality in their right proportion (45). While it is promising in making good use of alternative PBP sources such as CM in a particular range via nutrient balancing, previously conducted research has illustrated that the supplementation of EAA like lysine, methionine, and tryptophan to balance the AA profiles in CM enhances growth as well as improvement in the whole body composition (17, 22, 26, 59). Correspondingly, the plethora of research available on FM replacement with partial or full PBP supports the supplementation of EAA (mainly lysine, methionine, and threonine) to aid in achieving favourable AA profiles and improve palatability (59, 60). In the present study, we supplemented lysine, methionine, threonine, and leucine EAA to balance CM’s AA profiles.

At the end of the 56-day feeding trial, a significant improvement in the growth performance (FW, WGR, SGR, FCR HSI, VSI, ISI, and ILI) was witnessed in fish fed at the 4% replacement level in comparison with the other groups. Nonetheless, the fish fed with the highest replacement level (CM20 group) showed the worst growth performance. Similarly, dietary supplementation of CM above 8% (27) or 10% (26) reveals a reduction in feed intake, which causes a decrease in growth. Increasing the inclusion of CM in diets resulted in an increase in ricinine contents which led to a reduction in feed intake and growth performance in rainbow trout (*Oncorhynchus mykiss*) (61) and grass carp (*Ctenopharyngodon idellus*) as against lower CM replacement [40 g/kg feed (4%)] which had lesser ricinine content (< 20 mg/kg) (32). The supplementation of CM in broiler diets led to lower feed intake, poor FCR, and PER (62, 63), and even 83% mortality in growing chicks (64). The reduction in growth could be attributed to the higher contents of ANFs such as ricin, lectins, allergen, and ricinine in the diets. Although the contents of ANFs such as ricinine in feed were not analysed in the current study, the reduction in the growth performance could be attributed to the higher levels of ricinine content. More to this explanation is the metabolomics analysis which revealed the intensity of ricinine metabolite as being significantly high in the CM20 group than in the others.

Digestive enzyme activities, including amylase (AMS), trypsin (TRP), and lipase (LPS), aid in the breaking down of food for nutrient absorption (65, 66). The current study revealed a significantly high LPS activity in the CM groups compared to the other groups. No significant differences in the TRP and AMS were observed after FM’s dietary replacement with CM. A previous report shows a significant decrease in the activities of intestinal TRP and chymotrypsin (C-TRP) and liver TRP, C-TRP, AMS, and LPS after replacing FM with soybean meal in red seabream (*Pagrus major*) which was not in agreement with our findings (67). The digestive enzyme contents produced are undoubtedly regulated by the fish type, age, and diet (68, 69). Thus, the inconsistency can be attributed to the dietary CM supplement used in the current study since there is limited information on the subject matter.

Fish rely mainly on their non-specific and innate immunity in dealing with pathogenic invasion or the presence of toxins. Immune and antioxidant enzymes including C3, C4, IgM, LYZ, SOD, and GSH-Px help in host defence functions for growth and development as they can reflect the stress responses of fish due to infectious agents or toxins (70, 71). Complement is mainly responsible for the destruction and elimination of toxins. C3 and C4 are primarily produced by hepatocytes which can be activated to participate in immune response (72). IgM plays a vital role in bacterial opsonisation, toxin, and virus neutralisation, making them liable to phagocyte destruction in the host organism (73, 74). As part of the innate immune system, the LYZ functions by attacking, hydrolysing, and breaking glycosidic bonds in the peptidoglycan (75). In this study, FM replacement with CM significantly increased the C3, C4, IgM, and LYZ in the DI of juvenile hybrid groupers, indicating that CM substitution in diet could improve immunity, consistent with other reported studies (12, 76, 77). Contrarily, Zhang et al. (78), after substituting FM with soybean meal, showed significantly lower (*P* < 0.05) IgM, C3, and C4 levels in the intestine in comparison to the control. However, no significant differences were found in the LYZ enzyme activity among the treated groups (78). Optimum threonine (79) and leucine (80) in grass carp (*Ctenopharyngodon idella*) and black carp (*Mylopharyngodon piceus*), respectively, has been shown to significantly increase LYZ, C3, C4, and IgM immune parameters. Also, acute toxicity testing using chlorpyrifos in common carp (*Cyprinus carpio*) showed an LYZ increase in serum, hepatopancreas, and kidney, as well as an increase in serum and kidney IgM (81). Although this is the first report to reveal the effects of replacing FM with CM, the reason for the discrepancy can be ascribed to the supplementation of threonine and leucine AA in our study to achieve an optimum AA profile. Again, the changes could be attributed to the clearance and contributing effect of inflammatory reactions such that phagocytic cells are attracted to injury sites which can ultimately lyse pathogenic cells (75, 82, 83). We entreat further studies to explain the effects of CM on the non-specific immune systems of fish. For the antioxidant enzymes, while SOD is reported to support catalysing reactive O^–2^ to H_2_O_2_ partitioning (84), GSH-Px primarily shows the detoxification of hydrogen peroxides, as well as other peroxides such as lipid hydroperoxides (85). An increase in antioxidant enzyme implies that the antioxidant defence against reactive oxygen and free radical reaction is high; hence, the SOD and GSH-Px increase in the hybrid grouper’s intestine after replacing FM with CM supplementation suggests an improvement in the antioxidant status. The CM20 group exhibited the highest SOD and GSH-Px which was significantly higher than the results obtained for the control group. This phenomenon may be because of the high presence of monoterpene metabolites identified in the current study, such as the α-Pinene-2-oxide whose intensity was very high in the CM groups (CM20 obtaining the highest) than in the control. Pre-administration with α-Pinene-2-oxide sheltered U373-MG cells from stimulated oxidative damage of H_2_O_2_ via blocking the loss of cell viability (IC50: 79.70 mM to α-Pinene-2-oxide), which in turn prevented the formation of ROS and lipid peroxidation. As a result, there was a gross enhancement of endogenous antioxidant status via enhancing glutathione, SOD, CAT, GR, GSH-Px activities, HO-1 properties, and protein expression as reported by Porres-Martinez et al. (86). Thus the increase in the intensities of α-Pinene-2-oxide in the gut might have triggered the increase in the levels of SOD and GSH-Px observed in the present study.

The intestine is an important organ for digestion and absorption of nutrients. It plays an ardent role in regulating immunity, mucosa barrier, signal recognition, and the production of endogenous active molecules (87). Inducing enteritis has been one of the fascinating areas of studies that are now commonly used as the benchmark for the study of intestinal inflammation in fish, especially after FM is replaced with PBPs, including CM (18, 88). Fish physiology has been reported to improve along with the changes in the intestinal structure. Due to the high presence of ANFs in most PBP diets, the histopathological changes it comes with have been extensively researched. There is usually a swelling of the lamina propria (making their width bigger) and subepithelial mucosa, reduction in epithelial villi height, villi width, loss of normal enterocyte supranuclear absorptive vacuolisation, and infiltration of inflammatory cells. This, in a way, decreases the DI capacity to break down food into smaller particles, digest them and absorb the nutrients (18, 45, 88–91). Research accentuates that broader or taller epithelial villi and wider crypt depth are indications of higher absorption of nutrients in the gut (92). Although very little, other studies have also highlighted the histopathological changes of animals after CM supplementation, of which its severity is premised on the inclusion level. Diniz et al. (26), after 10% dietary CM supplementation, revealed an intense inflammation of the abomasums and intestines with corrosion of mucous membranes in cattle. Aslani et al. (27) observed hepatic necrosis, kidney acute tubular necrosis gastroenteritis, cardiac haemorrhage, and necrosis in sheep. Nagalakshm and Dhanalaksh (16) also revealed pathological lesions in the kidney, liver, intestines, and lungs in lambs after 10% CM as a result of the ANFs. On the contrary, an 8% supplementation level was purported to show no significant differences in the histopathological changes (28). There are some discrepancies looking at the results of previous studies. In the present study, the higher substitution of CM (CM20) in the diet caused CM-enteritis in the DI histological examination conducted by the AB-PAS staining and the SEM analysis. There was a significant decrease in the VH, VW, CD, and GC counts, as well as an increase in the MT and LP width in the CM20 group as compared to the other groups similar to the observation made previously in soybean enteritis (78). For the SEM results, there were fewer and weaker mucosal villi density, as well as villi atrophy (flattening or blunting), and this together might have caused the reduction in growth performance as the intestines were not tall and wider enough to absorb the needed nutrients for growth development. To the best of our knowledge, this is the first report to be conducted using a fish model (hybrid grouper) to assess the histopathological changes in the DI tissue by the AB-PAS staining and SEM after dietary CM supplementation, thus, we entreat that further studies be conducted.

Metabolomics has been effectively used to identify key potential metabolic biomarkers in the DI tissue of grouper after replacing FM with soybean meal (78). The application of LC-MS/MS analysis on four different types of castor bean revealed 60 key metabolites of high commercial value, which were all associated with primary and secondary metabolism, including fatty acids, amino acids, flavones, flavonol, flavanones, dopamine, and phenylpropanoids (93). Moreover, a recent report (94) identified *R*. *communis* to contain eighty-three metabolites, including alkaloids, flavonoids, terpenoids, derivatives of benzoic acid, tocopherols, coumarins, and fatty acids. However, it is vital to identify the key metabolites after their dietary supplement and know their intensities after digestion. It must be stated that, for the first time, this study labels the DI tissue’s metabolic profiling changes in juvenile grouper after being fed 4 and 20% CM protein replacement to FM using UPLC-MS, and the literature regarding UPLC-MS-based metabolomics on fish is rare. Compared with the current study, there were only 28 identified potential biomarkers.

Again, the present study discusses the biological relationships between the key potential biomarkers and their roles in intestinal health. Among the 28 potential biomarkers identified; 5 of them were organoheterocyclic compounds [Ricinine (sub-class: pyridinecarbonitriles), 4-Pyridoxic acid (sub-class: pyridinecarboxylic acids and derivatives), Butyryl fentanyl-d5 (sub-class: Fentanyl), L-beta-Imidazolelactic acid (sub-class: Imidazoles), and *N6*-Methyladenine (sub-class: Purines and purine derivatives)]; 7 belonged to Lipids and lipid-like molecules [10-Undecenoic acid (sub-class: Fatty acids and conjugates), α-Pinene-2-oxide (sub-class: Monoterpenoids), Linalool (sub-class: Monoterpenoids), Carvone (sub-class: Monoterpenoids), MAG (18:4) (sub-class: Lineolic acids and derivatives), 2-Hydroxymyristic acid (sub-class: Fatty acids and conjugates), and Taurocholic acid sodium salt hydrate (sub-class: Bile acids, alcohols and derivatives)]; 7 were in the super class of Organic Oxygen compounds (Jasmone (sub-class: Carbonyl compounds), Apocynin (sub-class: Carbonyl compounds), Dl-3-Hydroxy-kynurenine (sub-class: Carbonyl compounds), 3-[2-(β-D-Glucopyranosyloxy)-4-methoxyphenyl]propanoic acid (sub-class: Carbohydrates and carbohydrate conjugates), Sucrose (sub-class: Carbohydrates and carbohydrate conjugates), Stachyose (sub-class: Carbohydrates and carbohydrate conjugates), and α,α-Trehalose (sub-class: Carbohydrate and carbohydrate conjugates); 2 were in the super class of Benzenoids [2-(Formylamino)Benzoic Acid (sub-class: Benzoic acids and derivatives) and Styrene (sub-class: styrenes)]; 5 were in the super class Organic acids and derivatives [DL-α-Aminocaprylic acid (sub-class: Amino acids, peptides, and analogues), Levodopa (sub-class: Amino acids, peptides, and analogues), 12-Hydroxydodecanoic acid (sub-class: Medium-chain hydroxyl acids and derivatives), Citrulline (sub-class: Amino acids, peptides and analogues), and 10-Hydroxydecanoic acid (sub-class: Medium-chain hydroxyl acids, and derivatives)]; 1 was from super class Phenylpropanoids and polyketides [Naringenin (sub-class: Flavans)]; and 1 was from the super class Nucleosides, nucleotides, and analogues [2′-Deoxyuridine-5-monophosphate (sub-class: Pyrimidine deoxyribonucleotides)] which corresponds to previously identified metabolites in castor (*R*. *communis*) (94–96).

Ricinine, a metabolite of the class pyridines, and derivatives are considered a ricin toxin marker. Ricinine dosage varies but is found to be 2.3–32.9 g/kg in leaves, 3 g/kg in roots, 2.4 g/kg in stems, and 0.43–7.0 g.kg in its seeds (97, 98). Chickens are reported to be poisoned to death after consuming diets of 0.1 g/kg ricinine (99). 4-Pyridoxic acid (sub-class: pyridine carboxylic acids and derivatives) is the primary product of vitamin B_6_ in animals formed by the pyridoxal oxidation by a non-specific flavin adenine dinucleotide (FAD)-dependent aldehyde oxidase. Having a higher 4-Pyridoxic acid/pyridoxine ratio is linked with a deficiency in vitamin B_6_ (100, 101) which can affect intestinal morphology (decrease villi height and width) and absorption and metabolism of protein in animals (102). Butyryl fentanyl-d5 belongs to the class Piperidines (sub-class: Fentanyl), and Butyrates as dose-dependent is reported to promote intestinal barrier function at lower concentrations (≤2 mM) (103) but may disrupt intestinal barrier function at high concentrations (5 or 8 mM) by inducing apoptosis (104). Although a previous study reveals a higher concentration of L-beta-Imidazolelactic acid to be associated with an increase in antioxidant enzyme activities such as glutathione peroxidase (GPx), superoxide dismutase (SOD), and catalase (105) which are noted to play a vital role in the health of animals, the association of its relation with intestinal health is still unknown. Thus further research is warranted to explain such an association. This present study revealed significantly high intensities of Ricinine, 4-Pyridoxic acid, and Butyryl fentanyl-d5, and significantly low intensity of L-beta-Imidazolelactic acid in the higher replacement level as compared to the FM and the CM4 groups. Therefore the plausible reason for the witnessed disruption in the distal intestine can be attributed to the increase of Ricinine, 4-Pyridoxic acid, and Butyryl fentanyl-d5 intensities and decrease of L-beta-Imidazolelactic acid, which can also illustrate why higher replacement of FM with CM led to poorer growth performance.

The intensities of α-Pinene-2-oxide, Linalool, and Carvone were significantly increased with increasing replacement levels of CM. Monoterpenoids such as α-Pinene-2-oxide, Linalool, and Carvone are components of volatile essential oils from several plant products that are potent suppressors of plant growth (could be the reason for poor growth in the CM20 groups), but also cause a significant increase in antioxidant enzymes (106). Pre-treatment with α-Pinene-2-oxide inhibited ethanol-induced gastric lesions, reduced gastric juice volume and acidity, and increased gastric wall mucus in Swiss mice (107). α-Pinene-2-oxide is reported to stimulate oxidative damage of H_2_O_2_ through blocking the loss of cell viability, and prevents the formation of ROS and lipid peroxidation. Thus, gross enhancement of endogenous antioxidant status via enhancing glutathione, SOD, CAT, GR, GSH-Px activities, HO-1 properties, and protein expression exist (86). Various studies have described α-Pinene-2-oxide to display antimicrobial, anticancer, antitumor, anticancer, anti-inflammatory, and antiallergic properties. Nevertheless, most of these studies lack information concerning their relation to the intestinal health of animals (106); thus, more research is warranted in such areas.

The intensities of carbonyl compounds, including Jasmone, Apocynin, and Dl-3-Hydroxy-kynurenine, significantly increased with increasing replacement levels of CM to FM. In humans, intestinal ischemia usually occurs from impaired blood perfusion to the bowel due to various causes such as sepsis, cardiac insufficiency, vaso- and cardio-depressant drugs, and lifelong surgery, which in the long run progressively damages cell structures producing lesions that are further exacerbated (108). However, the treatment with Apocynin prevented such intestinal damage (109). Trehalose, a non-reducing disaccharide of glucose, is formed by stress conditions such as heat, oxidative stress, and other toxins. Based on previous findings, cytoplasmic trehalose accumulation protects the proteins and membranes from denaturation due to stress (110).

The main differential benzenoid metabolites belonged to Benzoic acids and derivatives and the Styrenes subclass. Benzoic acid irritates the human mucous membrane (111). Styrene is connected to several human diseases such as ulcerative colitis, non-alcoholic fatty liver disease, and the hereditary metabolic disorder celiac disease (112, 113). Reiko et al. (114), in an *in utero* exposure with Styrene, revealed a reduction in pub body growth and induced alteration in behaviour and neurotransmitter levels in brains. The significant increase in the intensities of 2-(Formylamino)Benzoic Acid and Styrene metabolites could be linked to the poor growth attained for grouper fish fed 20% CM replacement. Since studies relating to the association of 2-(Formylamino)Benzoic Acid and Styrene metabolites with the intestine health are limited, we recommend further studies to ascertain such relations.

The organic acid and derivatives are very large super-class with numerous pathways. Only the “Amino acids, peptides and analogues” and “Hydroxy acids and derivatives” sub-classes were discussed herein. Various amino acids, peptides, and analogues sub-class compounds have been known to down-regulate tyrosinase (TYR) gene expression or inhibit TYR catalytic activity. An example is Levodopa which is made via the biosynthesis from amino acid L-tyrosine by tyrosine hydroxylase enzyme (115). Also, L-arginine is an essential amino acid that is a precursor in synthesising Citrulline, proteins, urea, ornithine, creatinine, and agmatine, supporting the glutamate, proline, and polyamine metabolism at the whole organism level or cellular level in mammals. L-arginine’s availability and metabolisation can modulate inflammation, regulate the immune response to infections, and recover the physiological steady-state (116). Again, it has been long-established that hydroxy acids in crude extracts from plants have been used to treat diseases. The novel fatty acid, 12-Hydroxydodecanoic acid, was recently recognised as a metabolite with antifungal properties (117, 118). Citrulline has been involved in numerous regulatory roles, such as gut modulation, anti-inflammatory and antioxidative effects, protein synthesis, blood pressure regulation, nitrogen homeostasis, renal function, skeletal muscle function, cardiac function, and vascular health as well. The available information regarding the use of citrulline in animals is very limited; nevertheless, it is slightly gaining research interest as a result of its unique metabolism. Citrulline not only serve as functional marker for gut barrier dysfunction, but it has been associated also with several intestinal diseases, including short bowel syndrome, necrotizing enterocolitis and gastric ulcers (119). The observed increase in Citrulline metabolite intensity might have been due to the increased arginine content analysed (Supplementary Table 1) in the CM ingredient, which was higher than observed in the FM. Thus, there was modulation of inflammation and regulation of immune response, which sort of affirms why the immune enzyme activities analysed were higher than as obtained in the control group.

## Conclusion

In summary, the results in this study for the first time demonstrated the effects of replacing FM with CM on the growth, feed utilisation, immune response, digestive enzyme activities, and intestinal health of hybrid grouper (*Epinephelus fuscoguttatus*♀ × *Epinephelus lanceolatus*♂). A significant enhancement in terms of the parameters mentioned was achieved after replacing FM with 4% of CM. With the application of metabolomics, there were 28 identified metabolites as biomarkers of adherence to the CM diet. We propose further research to be conducted to validate the identified metabolites. In this study, the universal metabolomics platform (untargeted metabolomics) was used which only obtained relative estimates of the metabolites. Thus we recommend further studies which will deal with the use of targetted assays to obtain quantitative results for the identified potential biomarkers. Furthermore, it is necessary to conduct metabolomics profiling to determine how stable these potential biomarkers are when dealing with multiple time points or over an extended period and with a wider range of adherence to dietary CM patterns. The current study is limited to the dietary patterns of the CM trial, and it will be prudent to investigate the specificity of the potential biomarkers for the CM diets in comparison to other dietary patterns that vary in terms of macronutrients, such as lipids, proteins, and carbohydrates. The methods of using the integrated analysis of multi-omic technologies concerning different organs will be considered in our future work to further reveal the CM induced-enteritis mechanism.

## Data Availability Statement

The original contributions presented in the study are included in the article/Supplementary Material, further inquiries can be directed to the corresponding author/s.

## Ethics Statement

The animal study was reviewed and approved by the Animal Research and the Ethics Review Board of Guangdong Ocean University.

## Author Contributions

KA conceived and designed the experiment in consultation with X-HD, wrote the first draft of the manuscript which was later reviewed and criticized by X-HD and B-PT, and performed the animal experiment. S-YC, Q-HY, H-YL, and X-BY helped to collect the samples. KA, S-YC, Q-HY, H-YL, and X-BY analysed the data. X-HD and SZ aided in the purchasing of the re-agents. KA, X-HD, H-YL, Y-ZY, and HZ interpreted the statistical outcome. All authors consented to the submission of the manuscript for publication.

## Conflict of Interest

The authors declare that the research was conducted in the absence of any commercial or financial relationships that could be construed as a potential conflict of interest.

## Publisher’s Note

All claims expressed in this article are solely those of the authors and do not necessarily represent those of their affiliated organizations, or those of the publisher, the editors and the reviewers. Any product that may be evaluated in this article, or claim that may be made by its manufacturer, is not guaranteed or endorsed by the publisher.

## Footnotes

1
https://www.mzcloud.org/

2
http://www.chemspider.com

3
https://www.genome.jp/kegg/pathway.html

4
https://hmdb.ca/metabolites

5
http://www.lipidmaps.org/

## Supplementary Material

Supplementary Figure 1 The individual sample repeats representative UPLC-MC spectra of the distal intestine in the **(A)** positive, and **(B)** negative modes. FM-1 to FM-6 represent the individual sample repeats of FM; CM4-1 to CM4-6 represent the individual sample repeats of CM4; CM20-1 to CM20-6 represent the individual sample repeats of CM20 FM, fish meal (control group); CM4, 4% Castor meal (CM) protein replacement to FM protein; CM20, 20% CM protein replacement to FM protein.

Supplementary Figure 2 The results of the total PLS-DA score plots from the UPLC-MS spectra of the distal intestinal contents in positive **(A)** and negative **(B)** modes. A1 and A2 are, respectively the positive modes between FM and CM4, and FM and CM20 groups; B1 and B2 are respectively the negative modes between FM and CM4, and FM and CM20 groups. N = 6.

Supplementary Figure 3 The detailed clustering heatmap of total differential metabolites in the positive **(A)** and negative **(B)** modes after fishmeal replacement with castor meal. Individual groups are clustered in the vertical part, whereas those of the metabolites are clustered in the horizontal part. Colour intensity indicates the intensity of the metabolite. The relationship of metabolite content clustering between groups can be seen horizontally. The shorter the cluster branch is, the higher the similarity is. FM, fish meal (control group); CM4, 4% castor meal (CM) protein replacement to FM protein; CM20, 20% CM protein replacement to FM protein.
