# Supplementary material for: Ultra-Performance Liquid Chromatography-Mass Spectrometry-Based Untargeted Metabolomics Reveals the Key Potential Biomarkers for Castor Meal-Induced Enteritis in Juvenile Hybrid Grouper (Epinephelus fuscoguttatus♀ × E. lanceolatus♂)
Source: Front Nutr. 2022 Jun 16;9:847425. doi: 10.3389/fnut.2022.847425 (PMC9261911; doi:10.3389/fnut.2022.847425)
Supplement: Supplementary Figure 1 — The individual sample repeats representative UPLC-MC spectra of the distal intestine in the (A) positive, and (B) negative modes. FM-1 to FM-6 represent the individual sample repeats of FM; CM4-1 to CM4-6 represent the individual sample repeats of CM4; CM20-1 to CM20-6 represent the individual sample repeats of CM20 FM, fish meal (control group); CM4, 4% Castor meal (CM) protein replacement to FM protein; CM20, 20% CM protein replacement to FM protein. [file Data_Sheet_1.zip › Supplementary Tables/Supplementary Table S2.docx]

**Supplementary Table S2.** Amino acid composition of the experimental diets (% dry matter)

| AA^a^ | FM | CM4 | CM20 |
| --- | --- | --- | --- |
| EAA^b^ |  |  |  |
| Methionine | 0.97 | 1.09 | 1.01 |
| Lysine | 3.10 | 3.08 | 2.68 |
| Threonine | 1.84 | 1.82 | 1.67 |
| Leucine | 3.60 | 3.51 | 3.37 |
| Isoleucine | 2.02 | 1.99 | 1.98 |
| Valine | 2.23 | 2.27 | 2.24 |
| Phenylalanine | 2.17 | 2.07 | 1.98 |
| Histidine | 1.22 | 1.31 | 1.20 |
| Arginine | 2.60 | 2.67 | 3.06 |
| NEAA^c^ |  |  |  |
| Aspartate | 3.84 | 3.88 | 3.70 |
| Serine | 1.89 | 1.93 | 1.90 |
| Glutamate | 8.61 | 9.04 | 9.31 |
| Glycine | 2.30 | 2.33 | 2.08 |
| Alanine | 2.52 | 2.50 | 2.23 |
| Cystine | 0.50 | 0.56 | 0.54 |
| Proline | 2.69 | 2.88 | 2.55 |
| Tyrosine | 1.57 | 1.45 | 1.41 |
| ΣAA | 43.69 | 44.39 | 42.91 |
| ΣEAA | 19.75 | 19.81 | 19.19 |
| ΣNEAA | 23.92 | 24.57 | 23.72 |

^a^ AA: amino acid.

^b^ EAA: essential amino acids, tryptophan (Trp) content was not analysed in this study.

^c^ NEAA: non-essential amino acids.
