# Supplementary material for: Ultra-Performance Liquid Chromatography-Mass Spectrometry-Based Untargeted Metabolomics Reveals the Key Potential Biomarkers for Castor Meal-Induced Enteritis in Juvenile Hybrid Grouper (Epinephelus fuscoguttatus♀ × E. lanceolatus♂)
Source: Front Nutr. 2022 Jun 16;9:847425. doi: 10.3389/fnut.2022.847425 (PMC9261911; doi:10.3389/fnut.2022.847425)
Supplement: Supplementary Figure 1 — The individual sample repeats representative UPLC-MC spectra of the distal intestine in the (A) positive, and (B) negative modes. FM-1 to FM-6 represent the individual sample repeats of FM; CM4-1 to CM4-6 represent the individual sample repeats of CM4; CM20-1 to CM20-6 represent the individual sample repeats of CM20 FM, fish meal (control group); CM4, 4% Castor meal (CM) protein replacement to FM protein; CM20, 20% CM protein replacement to FM protein. [file Data_Sheet_1.zip › Supplementary Tables/Supplementary Table S3.docx]

**Supplementary Table S3.** The co-contained differential metabolites in the DI tissues in positive and negative mode

| Metabolite | FC1 | VIP1 | FC2 | VIP2 | Log2FC1 | Log2FC2 | Level |
| --- | --- | --- | --- | --- | --- | --- | --- |
| In positive mode |  |  |  |  |  |  |  |
| 10-Undecenoic acid | 0.238843 | 1.508296 | 0.086905 | 1.842013 | -2.06587 | -3.52442 | Down |
| Jasmone | 0.270643 | 1.484186 | 0.108873 | 1.832003 | -1.88554 | -3.19928 | Down |
| Apocynin | 0.357546 | 1.275985 | 0.126677 | 1.827533 | -1.4838 | -2.98078 | Down |
| α-Pinene-2-oxide | 0.431286 | 1.347646 | 0.207307 | 1.827753 | -1.21328 | -2.27016 | Down |
| DL- α-Aminocaprylic acid | 0.139044 | 1.333372 | 0.023061 | 1.844221 | -2.84639 | -5.43838 | Down |
| Linalool | 0.279886 | 1.21765 | 0.074878 | 1.824744 | -1.83709 | -3.73931 | Down |
| Butyryl fentanyl-d5 | 0.313224 | 1.353778 | 0.105068 | 1.854354 | -1.67473 | -3.25061 | Down |
| MAG (18:4) | 0.403432 | 1.098498 | 0.130114 | 1.812004 | -1.3096 | -2.94216 | Down |
| 3-(3-methylbut-2-en-1-yl)-3H-purin-6-amine | 0.308249 | 1.278233 | 0.110296 | 1.626473 | -1.69783 | -3.18055 | Down |
| Levodopa | 0.414501 | 1.072474 | 0.137043 | 1.815278 | -1.27055 | -2.86729 | Down |
| Styrene | 0.417555 | 1.333694 | 0.214159 | 1.767711 | -1.25996 | -2.22324 | Down |
| (+/-)12(13)-DiHOME | 0.415739 | 1.068778 | 0.139638 | 1.774074 | -1.26625 | -2.84024 | Down |
| 2-morpholino-1-phenyl-1-ethanol | 2.086802 | 1.049412 | 4.52596 | 1.655897 | 1.061294 | 2.178224 | Up |
| Nicotinuric acid | 0.138424 | 1.187075 | 0.016787 | 1.735555 | -2.85284 | -5.8965 | Down |
| Homosildenafil | 2.236411 | 1.012706 | 4.671348 | 1.583962 | 1.161185 | 2.223839 | Up |
| D-Galactosamine | 2.281222 | 1.116201 | 5.670388 | 1.704229 | 1.189807 | 2.503447 | Up |
| Beta-Muricholic acid | 0.225236 | 1.324061 | 0.066452 | 1.720219 | -2.15049 | -3.91154 | Down |
| Prolylglycine | 1.678039 | 1.149253 | 2.833461 | 1.680978 | 0.746776 | 1.502565 | Up |
| 3-hydroxy-4-(3-hydroxyphenyl)-1,2-dihydroquinolin-2-one | 2.439529 | 1.06022 | 6.804092 | 1.701136 | 1.286603 | 2.766403 | Up |
| 8(R)-Hydroxy-(5Z,9E,11Z,14Z)-eicosatetraenoic acid | 0.303788 | 1.199496 | 0.106881 | 1.645739 | -1.71886 | -3.22593 | Down |
| Glycolithocholic acid | 0.246757 | 1.072014 | 0.043925 | 1.697367 | -2.01884 | -4.50882 | Down |
| 10-Nitrolinoleate | 0.30554 | 1.152097 | 0.096089 | 1.78121 | -1.71057 | -3.37948 | Down |
| PLK | 2.133464 | 1.023739 | 4.340716 | 1.528502 | 1.093198 | 2.117933 | Up |
| Leukotriene C4 | 0.362822 | 1.084693 | 0.126484 | 1.537995 | -1.46267 | -2.98297 | Down |
| 7-(1-pyrrolidinyl)pyrimido[4,5-d]pyrimidin-4-amine | 1.818026 | 1.042021 | 3.232261 | 1.538485 | 0.862373 | 1.692544 | Up |
| 7Z, 10Z, 13Z, 16Z, 19Z-docosapentaenoic acid | 0.365105 | 1.477366 | 0.143612 | 1.704348 | -1.45362 | -2.79975 | Down |
| LPK | 1.606587 | 1.02166 | 3.247035 | 1.622946 | 0.683999 | 1.699123 | Up |
| 5,6-dimethoxy-2-(2-methoxyphenyl)-4H-chromen-4-one | 3.366268 | 1.157627 | 12.40873 | 1.636474 | 1.75115 | 3.633284 | Up |
| Galantamine | 2.230515 | 1.10934 | 5.115298 | 1.605422 | 1.157377 | 2.354818 | Up |
| THJ2201 N-(5-hydroxypentyl) metabolite | 1.990404 | 1.005355 | 4.529056 | 1.543504 | 0.993061 | 2.17921 | Up |
| Folic acid | 3.223069 | 1.030801 | 14.1152 | 1.621285 | 1.688435 | 3.819178 | Up |
| GQH | 3.088138 | 1.110127 | 20.99715 | 1.659879 | 1.626737 | 4.392121 | Up |
| Phe-Pro | 1.829246 | 1.051085 | 3.904578 | 1.506179 | 0.871249 | 1.965167 | Up |
| 6-(3-hydroxybutan-2-yl)-5-(hydroxymethyl)-4-methoxy-2H-pyran-2-one | 1.733686 | 1.116004 | 3.388774 | 1.482755 | 0.793843 | 1.760763 | Up |
| In negative mode |  |  |  |  |  |  |  |
| 12-Hydroxydodecanoic acid | 0.120205 | 1.419162 | 0.027767 | 1.81853 | -3.05644 | -5.17048 | Down |
| Dl-3-Hydroxy-kynurenine | 0.077799 | 1.517502 | 0.016487 | 1.837089 | -3.68411 | -5.9225 | Down |
| Naringenin | 0.175535 | 1.338141 | 0.041605 | 1.859708 | -2.51017 | -4.58709 | Down |
| Sucrose | 4.309948 | 1.292146 | 15.74781 | 1.738597 | 2.107671 | 3.97708 | Up |
| 2-Hydroxymyristic acid | 0.256684 | 1.138043 | 0.068743 | 1.762127 | -1.96194 | -3.86263 | Down |
| 10-Hydroxydecanoic acid | 0.276877 | 1.019155 | 0.05625 | 1.837494 | -1.85268 | -4.152 | Down |
| Linoleoyl Ethanolamide | 0.318955 | 1.290893 | 0.125391 | 1.781733 | -1.64857 | -2.99549 | Down |
| Mycophenolic acid | 0.202116 | 1.006902 | 0.027128 | 1.756113 | -2.30675 | -5.20405 | Down |
| Glycodeoxycholic Acid (hydrate) | 3.996706 | 1.169916 | 12.27504 | 1.633314 | 1.998811 | 3.617655 | Up |
| FAHFA (20:5/18:1) | 0.152483 | 1.263087 | 0.034065 | 1.676059 | -2.71328 | -4.87558 | Down |
| Taurocholic acid sodium salt hydrate | 4.663022 | 1.233674 | 16.11707 | 1.60119 | 2.221265 | 4.010518 | Up |
| N-Acetylmannosamine | 0.64275 | 1.017136 | 0.39708 | 1.620567 | -0.63767 | -1.3325 | Down |
| L-beta-Imidazolelactic acid | 1.56879 | 1.165759 | 2.493953 | 1.595224 | 0.649652 | 1.318434 | Up |
| D-Glucosamine 6-phosphate | 1.785479 | 1.002689 | 3.277594 | 1.503428 | 0.836311 | 1.712637 | Up |
| Nonadecanoic acid | 0.527576 | 1.052629 | 0.258705 | 1.516651 | -0.92255 | -1.95062 | Down |
| Ozagrel | 0.308395 | 1.010532 | 0.118129 | 1.531764 | -1.69715 | -3.08157 | Down |
| (5-L-Glutamyl)-L-Amino Acid | 1.786818 | 1.130856 | 3.455939 | 1.44108 | 0.837392 | 1.789078 | Up |

Note: FC, fold change; VIP, Variable Importance in the Projection. FC1, VIP1 and Log2FC1 were measured from DI tissue samples (CM4/FM group); FC2, VIP2 and Log2FC2 were measured from DI tissue samples (CM20/FM group); FM, fish meal (control group); CM4, 4% castor meal (CM) protein replacement to FM protein; CM20, 20% castor meal (CM) protein replacement to FM protein. Up: significant increase of metabolite; Down: significant decrease of metabolite.
