# Supplementary figures and images for: Ultra-Performance Liquid Chromatography-Mass Spectrometry-Based Untargeted Metabolomics Reveals the Key Potential Biomarkers for Castor Meal-Induced Enteritis in Juvenile Hybrid Grouper (Epinephelus fuscoguttatus♀ × E. lanceolatus♂)
Source: Front Nutr. 2022 Jun 16;9:847425. doi: 10.3389/fnut.2022.847425 (PMC9261911; doi:10.3389/fnut.2022.847425)

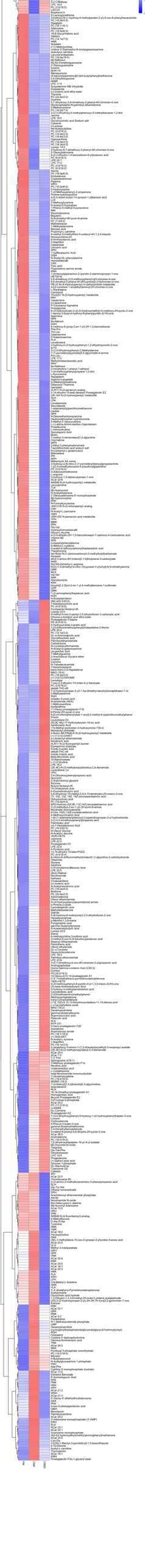

# B

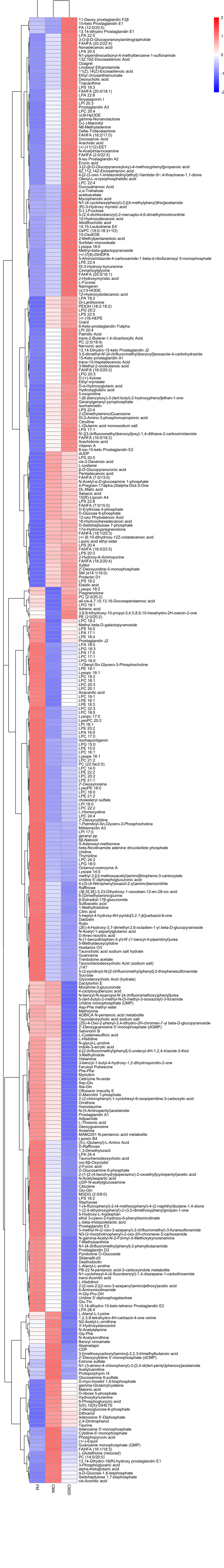

Supplement: Supplementary Figure 1 — The individual sample repeats representative UPLC-MC spectra of the distal intestine in the (A) positive, and (B) negative modes. FM-1 to FM-6 represent the individual sample repeats of FM; CM4-1 to CM4-6 represent the individual sample repeats of CM4; CM20-1 to CM20-6 represent the individual sample repeats of CM20 FM, fish meal (control group); CM4, 4% Castor meal (CM) protein replacement to FM protein; CM20, 20% CM protein replacement to FM protein. [file Data_Sheet_1.zip › Supplementary Figures/Supplementary Figure S3.pdf]
