# Supplementary material for: Ultra-Performance Liquid Chromatography-Mass Spectrometry-Based Untargeted Metabolomics Reveals the Key Potential Biomarkers for Castor Meal-Induced Enteritis in Juvenile Hybrid Grouper (Epinephelus fuscoguttatus♀ × E. lanceolatus♂)
Source: Front Nutr. 2022 Jun 16;9:847425. doi: 10.3389/fnut.2022.847425 (PMC9261911; doi:10.3389/fnut.2022.847425)
Supplement: Supplementary Figure 1 — The individual sample repeats representative UPLC-MC spectra of the distal intestine in the (A) positive, and (B) negative modes. FM-1 to FM-6 represent the individual sample repeats of FM; CM4-1 to CM4-6 represent the individual sample repeats of CM4; CM20-1 to CM20-6 represent the individual sample repeats of CM20 FM, fish meal (control group); CM4, 4% Castor meal (CM) protein replacement to FM protein; CM20, 20% CM protein replacement to FM protein. [file Data_Sheet_1.zip › Supplementary Tables/Supplementary Table S1.doc]

**Supplementary Table S1.** Amino acid composition of the experimental ingredients used in the current study (% dry matter)

| **Amino acid** | **Fish meal^a^** | **Castor meal^b^** |
| --- | --- | --- |
| Aspartate | 5.76 | 5.10 |
| Threonine | 2.79 | 1.99 |
| Serine | 2.55 | 3.09 |
| Glutamate | 8.53 | 11.17 |
| Glycine | 3.89 | 2.31 |
| Alanine | 4.21 | 2.21 |
| Cystine | 0.63 | 1.72 |
| Valine | 3.20 | 2.84 |
| Methionine | 1.83 | 0.73 |
| Isoleucine | 2.68 | 2.32 |
| Leucine | 4.73 | 3.30 |
| Tyrosine | 2.23 | 1.63 |
| Phenylalanine | 2.76 | 2.08 |
| Lysine | 4.97 | 1.67 |
| Histidine | 1.98 | 1.20 |
| Arginine | 3.70 | 6.33 |
| Proline | 2.49 | 1.86 |
| **Total** | **58.94** | **51.56** |

^a^ Supplied by China National Township Enterprises Corporation

^b^ Purchased from Shandong Weifang Supply and Marketing Industrial Co., Ltd., Shangdong, China.
