# Supplementary material for: Ultra-Performance Liquid Chromatography-Mass Spectrometry-Based Untargeted Metabolomics Reveals the Key Potential Biomarkers for Castor Meal-Induced Enteritis in Juvenile Hybrid Grouper (Epinephelus fuscoguttatus♀ × E. lanceolatus♂)
Source: Front Nutr. 2022 Jun 16;9:847425. doi: 10.3389/fnut.2022.847425 (PMC9261911; doi:10.3389/fnut.2022.847425)
Supplement: Supplementary Figure 1 — The individual sample repeats representative UPLC-MC spectra of the distal intestine in the (A) positive, and (B) negative modes. FM-1 to FM-6 represent the individual sample repeats of FM; CM4-1 to CM4-6 represent the individual sample repeats of CM4; CM20-1 to CM20-6 represent the individual sample repeats of CM20 FM, fish meal (control group); CM4, 4% Castor meal (CM) protein replacement to FM protein; CM20, 20% CM protein replacement to FM protein. [file Data_Sheet_1.zip › Supplementary Tables/Supplementary Table S12.doc]

**Supplementary Table S12.** The ten (10) most influential metabolites identified as the potential biomarkers in DI contents of CM4 vs. FM in positive mode (n = 6)

| Metabolite | Retention time (min) | Measured Mass (Da) | Calculated mass (Da) | Elemental composition | Scan mode | Log2FC | VIP |
| --- | --- | --- | --- | --- | --- | --- | --- |
| Ricinine^a,b^ | 6.566 | 164.0589 | 164.0586 | C_8_H_8_N_2_O_2_ | + | -8.51377 | 1.987896 |
| 10-Undecenoic acid^b^ | 14.118 | 184.1465 | 184.1463 | C_11_H_20_O_2_ | + | -2.06587 | 1.508296 |
| Jasmone^b^ | 14.111 | 164.1203 | **164.1201** | C_11_H_16_O | + | -1.88554 | 1.484186 |
| Apocynin^b^ | 6.582 | 166.0660 | 166.1739 | C_9_H_10_O_3_ | + | -1.4838 | 1.275985 |
| α-Pinene-2-oxide^b^ | 14.111 | 152.1202 | 152.2334 | C_10_H_16_O | + | -1.21328 | 1.347646 |
| 2-(Formylamino)Benzoic Acid^b^ | 6.345 | 165.0431 | 165.1461 | C_8_H_7_NO_3_ | + | -5.17974 | 1.929397 |
| 4-Pyridoxic acid^a,b^ | 6.339 | 183.0536 | 183.1614 | C_8_H_9_NO_4_ | + | -5.59463 | 1.947148 |
| DL-α-Aminocaprylic acid^a,b^ | 8.992 | 159.1262 | 159.2261 | C_8_H_17_NO_2_ | + | -2.84639 | 1.333372 |
| Linalool^b^ | 13.192 | 154.1360 | 154.2493 | C_10_H_18_O | + | -1.83709 | 1.21765 |
| Butyryl fentanyl-d5^b^ | 13.418 | 355.2725 | 355.5279 | C_23_H_25_[2]H_5_N_2_O | + | -1.67473 | 1.353778 |

^a^ The metabolite was confirmed by comparison to the metabolites of the mzCloud Database.

^b^ The metabolite was confirmed by comparison to the metabolites of the ChemSpider Database.

Note: FM, fish meal (control group); CM4, 4% castor meal protein replacement level to FM diet.
