# Supplementary material for: Ultra-Performance Liquid Chromatography-Mass Spectrometry-Based Untargeted Metabolomics Reveals the Key Potential Biomarkers for Castor Meal-Induced Enteritis in Juvenile Hybrid Grouper (Epinephelus fuscoguttatus♀ × E. lanceolatus♂)
Source: Front Nutr. 2022 Jun 16;9:847425. doi: 10.3389/fnut.2022.847425 (PMC9261911; doi:10.3389/fnut.2022.847425)
Supplement: Supplementary Figure 1 — The individual sample repeats representative UPLC-MC spectra of the distal intestine in the (A) positive, and (B) negative modes. FM-1 to FM-6 represent the individual sample repeats of FM; CM4-1 to CM4-6 represent the individual sample repeats of CM4; CM20-1 to CM20-6 represent the individual sample repeats of CM20 FM, fish meal (control group); CM4, 4% Castor meal (CM) protein replacement to FM protein; CM20, 20% CM protein replacement to FM protein. [file Data_Sheet_1.zip › Supplementary Tables/Supplementary Table S13.doc]

**Supplementary Table S13.** The ten (10) most influential metabolites identified as the potential biomarkers in DI contents of CM20 vs. FM in positive mode (n = 6)

| Metabolite | Retention time (min) | Measured Mass (Da) | Calculated mass (Da) | Elemental composition | Scan mode | Log2FC | VIP |
| --- | --- | --- | --- | --- | --- | --- | --- |
| Ricinine^a,b^ | 6.566 | 164.0589 | 164.1613 | C_8_H_8_N_2_O_2_ | + | -10.4643 | 1.761271 |
| Jasmone^b^ | 14.111 | 164.1203 | **164.1201** | C_11_H_16_O | + | -3.19928 | 1.832003 |
| α-Pinene-2-oxide^b^ | 14.111 | 152.1202 | 152.2334 | C_10_H_16_O | + | -2.27016 | 1.827753 |
| Levodopa^b^ | 7.104 | 197.0693 | 197.1879 | C_9_H_11_NO_4_ | + | -2.86729 | 1.815278 |
| 10-Undecenoic acid^b^ | 14.118 | 184.1465 | 184.1463 | C_11_H_20_O_2_ | + | -3.52442 | 1.842013 |
| Styrene^b^ | 14.108 | 104.0628 | 104.1491 | C_8_H_8_ | + | -2.22324 | 1.767711 |
| Carvone^a^ | 13.646 | 150.1046 | 150.1045 | C_10_H_14_O | + | -1.95556 | 1.814987 |
| Butyryl fentanyl-d5^b^ | 13.418 | 355.2725 | 355.5279 | C_23_H_25_[2]H_5_N_2_O | + | -3.25061 | 1.854354 |
| MAG (18:4)^b^ | 12.872 | 350.2461 | 350.4923 | C_21_H_34_O_4_ | + | -2.94216 | 1.812004 |
| DL-α-Aminocaprylic acid^a^ | 8.992 | 159.1262 | 159.1259 | C_8_H_17_NO_2_ | + | -5.43838 | 1.844221 |

^a^ The metabolite was confirmed by comparison to the metabolites of the mzCloud Database.

^b^ The metabolite was confirmed by comparison to the metabolites of the ChemSpider Database.

Note: FM, fish meal (control group); CM20, 20% castor meal protein replacement level to FM diet.
