# Supplementary material for: Ultra-Performance Liquid Chromatography-Mass Spectrometry-Based Untargeted Metabolomics Reveals the Key Potential Biomarkers for Castor Meal-Induced Enteritis in Juvenile Hybrid Grouper (Epinephelus fuscoguttatus♀ × E. lanceolatus♂)
Source: Front Nutr. 2022 Jun 16;9:847425. doi: 10.3389/fnut.2022.847425 (PMC9261911; doi:10.3389/fnut.2022.847425)
Supplement: Supplementary Figure 1 — The individual sample repeats representative UPLC-MC spectra of the distal intestine in the (A) positive, and (B) negative modes. FM-1 to FM-6 represent the individual sample repeats of FM; CM4-1 to CM4-6 represent the individual sample repeats of CM4; CM20-1 to CM20-6 represent the individual sample repeats of CM20 FM, fish meal (control group); CM4, 4% Castor meal (CM) protein replacement to FM protein; CM20, 20% CM protein replacement to FM protein. [file Data_Sheet_1.zip › Supplementary Tables/Supplementary Table S14.doc]

**Supplementary Table S14.** The ten (10) most influential metabolites identified as the potential biomarkers in DI contents of CM4 vs. FM in negative mode (n = 6)

| Metabolite | Retention time (min) | Measured Mass (Da) | Calculated mass (Da) | Elemental composition | Scan mode | Log2FC | VIP |
| --- | --- | --- | --- | --- | --- | --- | --- |
| 12-Hydroxydodecanoic acid^a,b^ | 11.863 | 216.1726 | 216.3172 | C_12_H_24_O_3_ | - | -3.05644 | 1.419162 |
| Dl-3-Hydroxy-kynurenine^a,b^ | 6.589 | 224.0798 | 224.2133 | C_10_H_12_N_2_O_4_ | - | -3.68411 | 1.517502 |
| Naringenin^a,b^ | 10.666 | 272.0686 | 224.2133 | C_15_H_12_O_5_ | - | -2.51017 | 1.338141 |
| 3-[2-(β-D-Glucopyranosyloxy)-4-methoxyphenyl]propanoic acid^a,b^ | 5.301 | 358.1268 | 358.3405 | C_16_H_22_O_9_ | - | -2.99202 | 1.560917 |
| 2'-Deoxyuridine-5-monophosphate^a,b^ | 1.425 | 308.0412 | 308.1819 | C_9_H_13_N_2_O_8_P | - | -2.20237 | 1.833838 |
| Citrulline^a,b^ | 1.302 | 175.0956 | 175.1857 | C_6_H_13_N_3_O_3_ | - | -1.51443 | 1.529026 |
| N6-Methyladenine^a,b^ | 6.531 | 149.0704 | 149.1533 | C_6_H_7_N_5_ | - | -1.51146 | 1.328207 |
| Sucrose^a,b^ | 1.409 | 342.1163 | 342.2965 | C_12_H_22_O_11_ | - | 2.107671 | 1.292146 |
| 2-Hydroxymyristic acid^a,b^ | 12.647 | 244.2038 | 244.3703 | C_14_H_28_O_3_ | - | -1.96194 | 1.138043 |
| 10-Hydroxydecanoic acid^a,b^ | 11.046 | 188.1413 | 188.2640 | C_10_H_20_O_3_ | - | -1.85268 | 1.019155 |

^a^ The metabolite was confirmed by comparison to the metabolites of the mzCloud Database.

^b^ The metabolite was confirmed by comparison to the metabolites of the ChemSpider Database.

Note: FM, fish meal (control group); CM4, 4% castor meal protein replacement level to FM diet.
