# Supplementary material for: Ultra-Performance Liquid Chromatography-Mass Spectrometry-Based Untargeted Metabolomics Reveals the Key Potential Biomarkers for Castor Meal-Induced Enteritis in Juvenile Hybrid Grouper (Epinephelus fuscoguttatus♀ × E. lanceolatus♂)
Source: Front Nutr. 2022 Jun 16;9:847425. doi: 10.3389/fnut.2022.847425 (PMC9261911; doi:10.3389/fnut.2022.847425)
Supplement: Supplementary Figure 1 — The individual sample repeats representative UPLC-MC spectra of the distal intestine in the (A) positive, and (B) negative modes. FM-1 to FM-6 represent the individual sample repeats of FM; CM4-1 to CM4-6 represent the individual sample repeats of CM4; CM20-1 to CM20-6 represent the individual sample repeats of CM20 FM, fish meal (control group); CM4, 4% Castor meal (CM) protein replacement to FM protein; CM20, 20% CM protein replacement to FM protein. [file Data_Sheet_1.zip › Supplementary Tables/Supplementary Table S15.doc]

**Supplementary Table S15.** The ten (10) most influential metabolites identified as the potential biomarkers in DI contents of CM20 vs. FM in negative mode (n =6)

| Metabolite | Retention time (min) | Measured Mass (Da) | Calculated mass (Da) | Elemental composition | Scan mode | Log2FC | VIP |
| --- | --- | --- | --- | --- | --- | --- | --- |
| Naringenin^a,b^ | 10.666 | 272.0686 | 272.2528 | C_15_H_12_O_5_ | - | -4.58709 | 1.859708 |
| Dl-3-Hydroxy-kynurenine^a,b^ | 6.589 | 224.0798 | 224.2144 | C_10_H_12_N_2_O_4_ | - | -5.9225 | 1.837089 |
| 12-Hydroxydodecanoic acid^a,b^ | 11.863 | 216.1726 | 216.3183 | C_12_H_24_O_3_ | - | -5.17048 | 1.81853 |
| Sucrose^a,b^ | 1.409 | 342.1163 | 342.2965 | C_12_H_22_O_11_ | - | 3.97708 | 1.738597 |
| 2-Hydroxymyristic acid^a,b^ | 12.647 | 244.2038 | 244.37032 | C_14_H_28_O_3_ | - | -3.86263 | 1.762127 |
| Taurocholic acid sodium salt hydrate^a^ | 10.52 | 538.2791 | 538.7000 | C_26_H_45_NNaO_7_S | - | 4.010518 | 1.60119 |
| Stachyose^b^ | 1.398 | 666.2229 | 666.5777 | C_24_H_42_O_21_ | - | 1.935064 | 1.621336 |
| 10-Hydroxydecanoic acid^a,b^ | 11.046 | 188.1413 | 188.2640 | C_10_H_20_O_3_ | - | -4.152 | 1.837494 |
| α,α-Trehalose^a,b^ | 12.321 | 342.1135 | 342.2965 | C_12_H_22_O_11_ | - | -4.28685 | 1.781023 |
| L-beta-Imidazolelactic acid^a,b^ | 1.091 | 156.0535 | 156.1393 | C_6_H_8_N_2_O_3_ | - | 1.318434 | 1.595224 |

^a^ The metabolite was confirmed by comparison to the metabolites of the mzCloud Database.

^b^ The metabolite was confirmed by comparison to the metabolites of the ChemSpider Database.

Note: FM, fish meal (control group); CM20, 20% castor meal protein replacement level to FM diet.
