# Supplementary material for: Ultra-Performance Liquid Chromatography-Mass Spectrometry-Based Untargeted Metabolomics Reveals the Key Potential Biomarkers for Castor Meal-Induced Enteritis in Juvenile Hybrid Grouper (Epinephelus fuscoguttatus♀ × E. lanceolatus♂)
Source: Front Nutr. 2022 Jun 16;9:847425. doi: 10.3389/fnut.2022.847425 (PMC9261911; doi:10.3389/fnut.2022.847425)
Supplement: Supplementary Figure 1 — The individual sample repeats representative UPLC-MC spectra of the distal intestine in the (A) positive, and (B) negative modes. FM-1 to FM-6 represent the individual sample repeats of FM; CM4-1 to CM4-6 represent the individual sample repeats of CM4; CM20-1 to CM20-6 represent the individual sample repeats of CM20 FM, fish meal (control group); CM4, 4% Castor meal (CM) protein replacement to FM protein; CM20, 20% CM protein replacement to FM protein. [file Data_Sheet_1.zip › Supplementary Tables/Supplementary Table S16.doc]

**Supplementary Table S16.** Intensities of metabolites identified as the potential biomarkers in DI contents of CM4, CM20 vs. FM in positive mode (n = 6)

| Group | Ricinine | 10 Undecenoic acid | Jasmone | Apocynin | α Pinene-2-oxide |
| --- | --- | --- | --- | --- | --- |
| FM | 5963129.50±410757.07^a^ | 6773559.83±343250.22^a^ | 16485177.20±753470.93^a^ | 1365719.33±81171.43^a^ | 16330311.17±732849.51^a^ |
| CM4 | 2342252011.80±146110995.78^b^ | 28359933.50±1642896.15^b^ | 54026732.00±1130470.28^b^ | 3819700.67±229622.15^b^ | 37864250.50±2094977.75^b^ |
| CM20 | 9461322982.75±579178551.84^c^ | 85933955.50±7716204.97^c^ | 156438819.00±11863282.29^c^ | 11649928.00±1142013.17^c^ | 78773450.50±4480124.12^c^ |

Note: Data represented the mean ± S.E (Turkey’s HSD, *P* < 0.05; *n* = 6). Means in the same columns with no superscripts do not differ significantly (*P* > 0.05; by Tukey’s HSD test) among groups. Where; FM = fishmeal; CM4 = 4% replacement of fishmeal with castor meal; and CM20 = 20% replacement of fishmeal with castor meal.

**Supplementary Table S16** **(continue).** Intensities of metabolites identified as the potential biomarkers in DI contents of CM4, CM20vs. FM in positive mode (n = 6)

| Group | 2-(Formylamino)Benzoic Acid | 4-Pyridoxic acid | DL-α-Aminocaprylic acid | Linalool | Butyryl fentanyl-d5 |
| --- | --- | --- | --- | --- | --- |
| FM | 1326185.17±65043.57^a^ | 2407101.00±111040.98^a^ | 716193.33±33169.33^a^ | 7463214.83±349569.58^a^ | 3254537.50±312102.39^a^ |
| CM4 | 35820943.25±3675913.04^a^ | 97982864.40±14463905.71^b^ | 5150839.17±564320.23^a^ | 26665150.67±2675665.16^a^ | 11621309.83±760345.23^b^ |
| CM20 | 167754497.75±30017421.65^b^ | 455164072.67±31710027.37^c^ | 32722719.17±4169191.51^b^ | 104671068.50±11625509.79^b^ | 36648207.83±1712666.82^c^ |

Note: Data represented the mean ± S.E (Turkey’s HSD, *P* < 0.05; *n* = 6). Means in the same columns with no superscripts do not differ significantly (*P* > 0.05; by Tukey’s HSD test) among groups. Where; FM = fishmeal; CM4 = 4% replacement of fishmeal with castor meal; and CM20 = 20% replacement of fishmeal with castor meal.

**Supplementary Table S16** **(continue).** Intensities of metabolites identified as the potential biomarkers in DI contents of CM4, CM20vs. FM in positive mode (n = 6)

| Group | Levodopa | Styrene | Carvone | MAG (18:4) |
| --- | --- | --- | --- | --- |
| FM | 649206.00±45683.49^a^ | 14850444.50±1181861.14^a^ | 10950131.00±448788.94^b^ | 4578138.33±389679.77^a^ |
| CM4 | 1566234.50±154540.53^a^ | 37231923.67±2706040.80^b^ | 0.00±0.00^a^ | 11681313.50±699539.66^b^ |
| CM20 | 4737227.33±406645.87^b^ | 69342935.83±5138592.68^c^ | 42471851.33±2695448.86^c^ | 36852385.67±2280388.72^c^ |

Note: Data represented the mean ± S.E (Turkey’s HSD, *P* < 0.05; *n* = 6). Means in the same columns with no superscripts do not differ significantly (*P* > 0.05; by Tukey’s HSD test) among groups. Where; FM = fishmeal; CM4 = 4% replacement of fishmeal with castor meal; and CM20 = 20% replacement of fishmeal with castor meal.
