# Supplementary material for: Ultra-Performance Liquid Chromatography-Mass Spectrometry-Based Untargeted Metabolomics Reveals the Key Potential Biomarkers for Castor Meal-Induced Enteritis in Juvenile Hybrid Grouper (Epinephelus fuscoguttatus♀ × E. lanceolatus♂)
Source: Front Nutr. 2022 Jun 16;9:847425. doi: 10.3389/fnut.2022.847425 (PMC9261911; doi:10.3389/fnut.2022.847425)
Supplement: Supplementary Figure 1 — The individual sample repeats representative UPLC-MC spectra of the distal intestine in the (A) positive, and (B) negative modes. FM-1 to FM-6 represent the individual sample repeats of FM; CM4-1 to CM4-6 represent the individual sample repeats of CM4; CM20-1 to CM20-6 represent the individual sample repeats of CM20 FM, fish meal (control group); CM4, 4% Castor meal (CM) protein replacement to FM protein; CM20, 20% CM protein replacement to FM protein. [file Data_Sheet_1.zip › Supplementary Tables/Supplementary Table S17.doc]

**Supplementary Table S17.** Intensities of metabolites identified as the potential biomarkers in DI contents of CM4, CM20 vs. FM in negative mode (n = 6)

| Group | 12-Hydroxydodecanoic acid | Dl-3-Hydroxy-kynurenine | Naringenin | 3-[2-(β-D-Glucopyranosyloxy)-4-methoxyphenyl]propanoic acid | 2'-Deoxyuridine-5-monophosphate |
| --- | --- | --- | --- | --- | --- |
| FM | 469388.33±31738.73^a^ | 116506.33±3934.40 | 1491904.00±92850.62 | 115291.67±5704.12^a^ | 2309531.17±252603.31^a^ |
| CM4 | 4071579.33±213554.20^a^ | 1697533.17±102238.62 | 30640581.83±14756295.59 | 807392.17±73848.17^a^ | 10629230.00±830053.67^b^ |
| CM20 | 15237764.00±1692078.65^b^ | 7066410.33±702108.03 | 156194833.83±73548491.47 | 3597385.33±378510.32^b^ | 17859152.33±1312358.92^c^ |

Note: Data represented the mean ± S.E (Turkey’s HSD, *P* < 0.05; *n* = 6). Means in the same columns with no superscripts do not differ significantly (*P* > 0.05; by Tukey’s HSD test) among groups. Where; FM = fishmeal; CM4 = 4% replacement of fishmeal with castor meal; and CM20 = 20% replacement of fishmeal with castor meal.

**Supplementary Table S17** **(continue).** Intensities of metabolites identified as the potential biomarkers in DI contents of CM4, CM20 vs. FM in negative mode (n=6)

| Group | Citrulline | N6-Methyladenine | Sucrose | 2-Hydroxymyristic acid | 10-Hydroxydecanoic_acid |
| --- | --- | --- | --- | --- | --- |
| FM | 2990019.33±251236.91^a^ | 330684.00±35983.03^a^ | 1454549449.17±110708871.55^b^ | 871037.33±59071.79^a^ | 615286.83±35438.80^a^ |
| CM4 | 9708702.00±282223.84^b^ | 942777.33±80292.03^b^ | 287486507.83±31721646.16^a^ | 3560093.00±441533.91^b^ | 2722236.50±294757.92^a^ |
| CM20 | 13728370.00±789211.59^c^ | 1903077.83±175911.44^c^ | 65698505.67±5642927.82^a^ | 14837511.33±891983.76^c^ | 12271718.67±1262149.75^b^ |

Note: Data represented the mean ± S.E (Turkey’s HSD, *P* < 0.05; *n* = 6). Means in the same columns with no superscripts do not differ significantly (*P* > 0.05; by Tukey’s HSD test) among groups. Where; FM = fishmeal; CM4 = 4% replacement of fishmeal with castor meal; and CM20 = 20% replacement of fishmeal with castor meal.

**Supplementary Table S17** **(continue).** Intensities of metabolites identified as the potential biomarkers in DI contents of CM4, CM20 vs. FM in negative mode (n=6)

| Group | Taurocholic acid sodium salt hydrate | Stachyose | α,α-Trehalose | L-beta-Imidazolelactic acid |
| --- | --- | --- | --- | --- |
| FM | 6618164.67±773569.87^b^ | 341363608.83±30817633.65^c^ | 159429.50±16611.55^b^ | 1568081.33±139669.34^b^ |
| CM4 | 1812841.17±195173.54^a^ | 0.00±0.00^a^ | 0.00±0.00^a^ | 999548.33±98097.87^a^ |
| CM20 | 502489.33±44752.06^a^ | 139269845.83±13090074.49^b^ | 5726626.00±860098.35^c^ | 628753.33±49450.21^a^ |

Note: Data represented the mean ± S.E (Turkey’s HSD, *P* < 0.05; *n* = 6). Means in the same columns with no superscripts do not differ significantly (*P* > 0.05; by Tukey’s HSD test) among groups. Where; FM = fishmeal; CM4 = 4% replacement of fishmeal with castor meal; and CM20 = 20% replacement of fishmeal with castor meal.
